# Pseudallenes A and B, new sulfur-containing ovalicin sesquiterpenoid derivatives with antimicrobial activity from the deep-sea cold seep sediment-derived fungus *Pseudallescheria boydii* CS-793

**DOI:** 10.3762/bjoc.20.42

**Published:** 2024-02-28

**Authors:** Zhen Ying, Xiao-Ming Li, Sui-Qun Yang, Hong-Lei Li, Xin Li, Bin-Gui Wang, Ling-Hong Meng

**Affiliations:** 1 CAS and Shandong Province Key Laboratory of Experimental Marine Biology, Center for Ocean Mega-Science, Institute of Oceanology, Chinese Academy of Sciences, Nanhai Road 7, Qingdao 266071, China; 2 University of Chinese Academy of Sciences, Yuquan Road 19A, Beijing 100049, China; 3 Laboratory of Marine Biology and Biotechnology, Qingdao National Laboratory for Marine Science and Technology, Wenhai Road 1, Qingdao 266237, China

**Keywords:** antimicrobial activity, cold seep, *Pseudallescheria boydii*, sesquiterpenoid

## Abstract

Pseudallenes A and B (**1** and **2**), the new and rare examples of sulfur-containing ovalicin derivatives, along with three known analogues **3**–**5**, were isolated and identified from the culture extract of *Pseudallescheria boydii* CS-793, a fungus obtained from the deep-sea cold seep sediments. Their structures were established by detailed interpretation of NMR spectroscopic and mass spectrometric data. X-ray crystallographic analysis confirmed and established the structures and absolute configurations of compounds **1**–**3**, thus providing the first characterized crystal structure of an ovalicin-type sesquiterpenoid. In the antimicrobial assays, compounds **1**–**3** showed broad-spectrum inhibitory activities against several plant pathogens with MIC values ranging from 2 to 16 μg/mL.

## Introduction

Marine cold seeps are typical of chemosynthetically driven ecosystems, characterized by methane-rich fluid emissions and unique sulfur oxidation–reduction reactions [1]. Due to the unique habitat, microorganisms surviving in the deep-sea cold seeps may serve as promising sources of secondary metabolites with functional and structural diversity [2]. In particular, two indole diketopiperazine alkaloids containing an unprecedented spiro[bicyclo[2.2.2]octane-diketopiperazine] skeleton, chevalinulins A and B, and the first cytochalasin homodimer containing a thioether bridge, verruculoid A, were identified from deep-sea cold seeps-derived fungi and were described to have potent proangiogenic and antimicrobial activities [3–4].

As part of our continuing search for bioactive metabolites from deep-sea-derived fungi [3–6], the fungal strain *Pseudallescheria boydii* CS-793, which was obtained from sediments collected at the deep-sea cold seep area in the Northeast of the South China Sea, attracted our attention. Several meroterpenoids, alkaloids, polyketides, and sesquiterpenoids from the species displayed various biological properties including anti-inflammatory, antimicrobial, and cytotoxic activities [7–9]. In the present work, two rare new examples of sulfur-containing ovalicin sesquiterpenoids (**1**, **2**), together with three known related analogs (**3**–**5**) [10–13] have been isolated and identified from the bioactive fraction of *P. boydii* CS-793. Details of the isolation and purification, structure elucidation, and biological evaluation of compounds **1**–**5** are described herein.

## Results and Discussion

**Figure 1:**
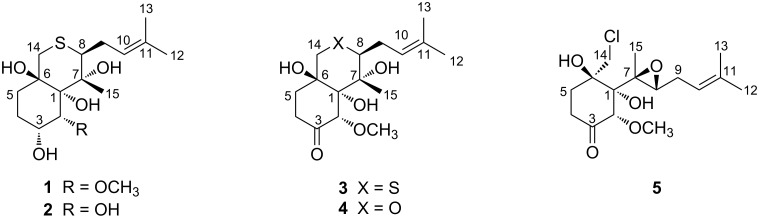
Chemical structures of compounds **1**–**5** isolated from *P. boydii* CS-793.

For chemical investigation, the solvent EtOAc was used to extract the fermentation culture of the fungus *P. boydii* CS-793 to afford an organic extract. Isolation and purification of the crude extract with a combination of column chromatography (CC) by Lobar LiChroprep RP-18, silica gel, Sephadex LH-20, and semi-preparative HPLC, yielded compounds **1**–**5** (Figure 1).

### Structure elucidations

Pseudallene A (**1**), initially obtained as colorless amorphous powder, was assigned a molecular formula of C_16_H_28_O_5_S with three indices of hydrogen deficiency according to the HRESIMS data. The ^1^H NMR spectrum (Table 1 and Figure S1 in Supporting Information File 1) for compound **1** showed well-dispersed signals over a wide field range, and aided by HSQC experiment, these signals were attributable to four singlet methyls [δ_Η_ 1.65 (3H, s, H_3_-12), 1.55 (3H, s, H_3_-13), 1.39 (3H, s, H_3_-15), and 3.37 (3H, s, 2-OMe)], four aliphatic methylenes, four methines (with two oxygenated and one olefinic), and four exchangeable protons. The ^13^C NMR spectroscopic data (Table 1) displayed all 16 resonances which were classified by DEPT experiments into the categories of four methyls (including one methoxy), four methylenes, four methines (with one olefinic and two oxygenated), and four non-protonated carbons (with one olefinic and three bonded to oxygen).

**Table 1:** ^1^H (500 MHz) and ^13^C NMR (125 MHz) data for compounds **1**–**3**.

no.	**1**		**2**		**3**	
	δ_C_^a^	δ_H_^b^, mult (*J* in Hz)	δ_C_^a^	δ_H_^b^, mult (*J* in Hz)	δ_C_^c^	δ_H_^d^, mult (*J* in Hz)

1	78.4, C		76.8, C		82.4, C	
2	79.2, CH	3.75 d (3.1)	75.9, CH	3.89 d (8.7)	87.4, CH	4.67, s
3	65.0, CH	4.22 dq (6.1, 3.1)	70.0, CH	3.57 ddd (11.1, 8.7, 5.5)	207.7, C	
4	26.8, CH_2_	α 1.61 overlap β 1.87 overlap	28.2, CH_2_	α 1.56 overlap β 1.60 overlap	36.7, CH_2_	α 2.78 td (13.7, 6.9) β 2.40 ddd (13.7, 5.4, 1.7)
5	30.0, CH_2_	α 1.03 dt (11.6, 2.7) β 1.97 overlap	33.7, CH_2_	α 1.09 ddd (13.2, 4.3, 2.7) β 1.77 td (13.2, 4.8)	35.4, CH_2_	α 1.72 ddd (13.5, 6.9, 1.7) β 2.26 td (13.5, 5.4)
6	73.5, C		72.7, C		73.1, C	
7	75.8, C		76.8, C		76.8, C	
8	45.8, CH	2.93 dd (11.5, 2.4)	45.7, CH	2.94 overlap	47.5, CH	2.91 dd (11.5, 2.5)
9	26.2, CH_2_	α 1.68 overlap β 2.55 dd (15.2, 6.3)	26.1, CH_2_	α 1.67 overlap β 2.53 overlap	26.6, CH_2_	α 1.85 ddd (14.8, 11.5, 7.9) β 2.63 dd (15.5, 6.3)
10	123.2, CH	5.14 t (6.9)	123.2, CH	5.12 t (6.4)	121.8, CH	5.22 t (7.3)
11	131.4, C		131.6, C		134.1, C	
12	25.5, CH_3_	1.65 s	25.6, CH_3_	1.65 s	26.0, CH_3_	1.75 s
13	17.6, CH_3_	1.55 s	17.8, CH_3_	1.55 s	18.1, CH_3_	1.64 s,
14	35.9, CH_2_	α 1.93 d (13.7) β 3.01 d (13.7)	36.1, CH_2_	α 1.92 d (13.6) β 2.95 overlap	37.5, CH_2_	α 2.20 d (13.9) β 3.27 d (13.9)
15	19.8, CH_3_	1.39 s	19.3, CH_3_	1.43 s	19.9, CH_3_	1.54 s
1-OH		5.28 s		3.93 s		
2-OCH_3_/OH	55.1, CH_3_	3.37 s		4.50 brs	59.3, CH_3_	3.55 s
3-OH		5.29 d (6.0)		5.61 brs		
6-OH		4.32 s		4.16 s		3.71 s
7-OH		4.79 s		3.34		4.28 s

^a^Data collected at 125 MHz in DMSO-*d*_6_. ^b^Data collected at 500 MHz in DMSO-*d*_6_. ^c^Data collected at 125 MHz in CDCl_3_. ^d^Data collected at 500 MHz in CDCl_3_.

**Figure 2:**
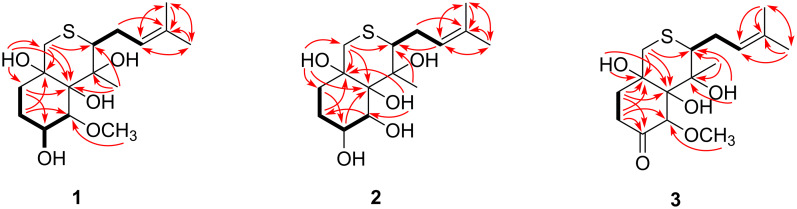
Key ^1^H-^1^H COSY (bond lines), and HMBC (red arrows) correlations of **1**–**3**.

Detailed interpretation of the COSY spectrum of compound **1** revealed the presence of two discrete proton spin-coupling systems corresponding to a –CH–CH(OH)–CH_2_–CH_2_– unit (C-2 to C-5), and a –CH–CH_2_–CH= moiety (C-8 to C-10) (Figure 2). HMBC correlations from H-4 to C-2 and C-6, from H-5 to C-1 and C-3, from 1-OH to C-2 and C-6, and from 6-OH to C-1, C-5, and C-14, led to the construction of the cyclohexane ring for **1**, while HMBC correlations from H-9 to C-11, and from H-12 and H-13 to C-10 and C-11 constructed the isopentenyl group. Further HMBC correlations from H-15 to C-1, C-7, and C-8 connected the cyclohexane ring and isopentenyl from C-1 and C-8 via the non-protonated carbon C-7. The assignment of the thio-ether bond between C-8 and C-14 was supported by the HMBC correlation from H-14 to C-8, as well as the molecular formula combined with chemical shifts (δ_C_ 35.9, CH_2_-14, and δ_C_ 45.8, CH-8) [12]. Furthermore, HMBC correlations from the protons of methoxy to C-2 attached the methoxy group to C-2.

**Figure 3:**
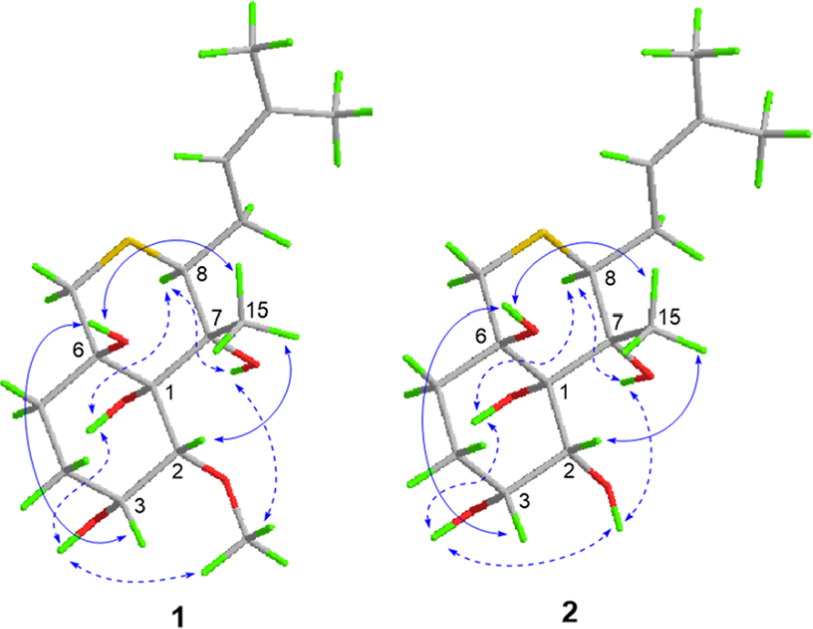
NOE correlations of compounds **1** and **2** (solid line indicates β-orientation and dashed lines represent α-orientation).

**Figure 4:**
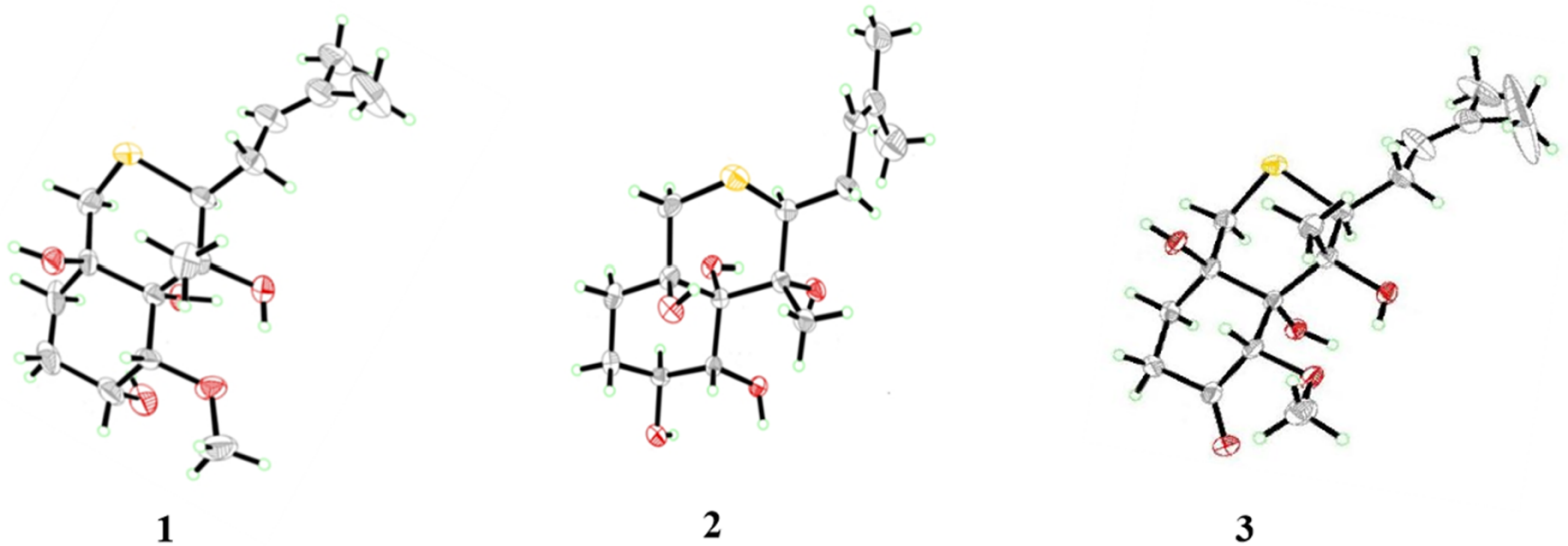
X-ray crystal structure of compounds **1**–**3** (with a thermal ellipsoid probability of 50%).

The relative configuration of compound **1** was determined by analysis of NOESY data (Figure 3). NOE cross peaks from H-15 to H-2, 6-OH, and from H-3 to 6-OH indicated the cofacial orientation of these groups, while correlations from 1-OH to H-8, 7-OH, 2-OMe, and 3-OH suggested the opposite position of these groups. To unambiguously clarify the structure of compound **1**, crystals suitable for X-ray crystal analysis were obtained by slow evaporation of the solvent, which could be analyzed by X-ray diffraction analysis using Cu Kα radiation (Figure 4). The resulting Flack parameter, 0.019(6), allowed the assignment of the absolute configurations of all the stereogenic centers in compound **1** as 1*R*, 2*R*, 3*R*, 6*R*, 7*R*, 8*S*. This is likely the first characterized crystal structure of an ovalicin-type sesquiterpenoid.

The molecular formula of pseudallene B (**2**) was assigned as C_15_H_26_O_5_S (three unsaturations), with one CH_2_ unit less than **1**, based on positive HRESIMS data. Its NMR spectroscopic data were similar to compound **1**. However, the obvious differences from compound **1** was the absence of the methoxy signals resonating at δ_C_/δ_H_ 55.1/3.37 (2-OCH_3_) in compound **2**, which suggested the replacement of an OCH_3_ in **1** by an OH in compound **2**. This deduction was further verified by the COSY and HMBC correlations (Figure 2).

The structure and relative configuration of compound **2** were deduced the same as for **1** by NOE correlations (Figure 3). Moreover, the absolute configurations of compound **2** were unambiguous determined by X-ray diffraction with the refined Flack parameter of 0.048(7), which suggested all the stereogenic centers in compound **2** as 1*R*, 2*R*, 3*R*, 6*R*, 7*R*, 8*S*.

In addition to compounds **1** and **2**, three related ovalicin-type sesquiterpenoid derivatives **3** [10], **4** [11], and chlovalicin (**5**) [12–13] were isolated and identified from the fungus *P. boydii* CS-793. It should be mentioned that compound **3** was the first sulfur-containing ovalicin sesquiterpenoid, which was previously isolated from *Sporothrix sp.* FO-4649, but its absolute configuration was not explicitly represented, and their ^1^H and ^13^C NMR data were incomplete [10]. Thus, a full assignment of the NMR data for compound **3** was conducted (Table 1) and its absolute configurations were assigned as 1*R*, 2*S*, 6*R*, 7*R*, 8*S* by single-crystal X-ray diffraction analysis with a Flack parameter of 0.024(6) (Figure 4).

**Scheme 1:**
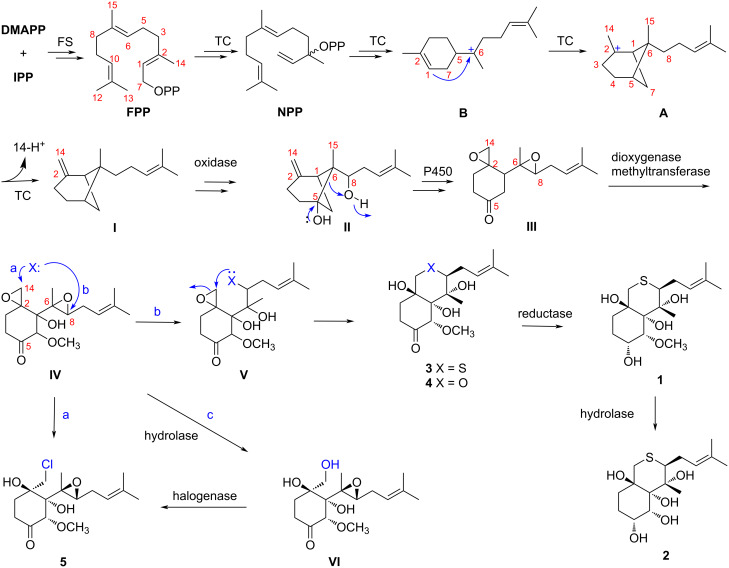
Proposed biosynthetic pathway for compounds **1**–**5**.

A plausible biosynthetic pathway for compounds **1**–**5** is proposed as shown in Scheme 1. In this pathway, the bergamotene sesquiterpenoid (**I**) is presumed to be a key intermediate cyclized from farnesyl diphosphate (FPP) via nerolidyl diphosphate (NPP) followed by a bisabolyl cation [14]. Subsequent oxidation (bishydroxylation) catalyzed by some oxygenase such as P450 would afford the key intermediate **II**, which could be transferred to **III** by cyclization and epoxidation. Oxidation and methylation of intermediate **III** would produce **IV**. Compounds **1**–**4** could be obtained by nucleophilic attack at C-8 with the hydroxy or thiol group from **IV** via intermediate **V**, followed by oxidation and cyclization (pathway b), while nucleophilic attack at C-14 of intermediate **IV** by a chloride could generate compound **5** (pathway a). In addition, compound **5** might also be derived from intermediate **IV** by cleavage of the ester bond at C-2 to form the intermediate **VI** [15], followed by chlorination (pathway c).

Compounds **1**–**3** were tested against seven human- and marine-derived aquatic pathogenetic bacteria (*Edwardsiella tarda*, *Escherichia coli*, *Micrococcus luteus*, *Pseudomonas aeruginosa*, *Vibrio anguillarum*, *Vibrio harveyi*, and *Vibrio vulnificus*), and six plant pathogenic fungi (*Alternaria brassicae*, *Colletotrichum gloeosporioides*, *Coniothyrium diplodiella*, *Curvularia spicifera*, *Fusarium proliferatum*, and *Penicillium digitatum*) (Table 2). In the antibacterial screening, none of the compounds displayed potent activity against the tested strains (MIC ≥ 32 μg/mL). The antifungal assays showed that compounds **1**–**3** exhibited potent activities against the plant pathogenic fungi. Compound **1** exhibited prominent activity against *F. proliferatum*, *C. spicifera*, *and C. gloeosporioides* with MIC values of 2, 4, and 4 μg/mL, respectively, while compound **3** showed considerable activities against *C. diplodiella*, *C. spicifera* and *F. proliferatum* with MIC values of 2, 4, and 4 μg/mL, respectively.

**Table 2:** Antimicrobial activities of compounds **1**–**3** (MIC).^a^

strains	**1**		**2**		**3**		positive control	
	μg/mL	μM	μg/mL	μM	μg/mL	μM	μg/mL	μM

*E*. *tarda*^b^	32	96.38	–	–	–	–	2	6.19
*V. anguillarum* ^b^	32	96.38	64	201.26	32	96.96	0.5	1.55
*V. harveyi* ^b^	–	–	–	–	–	–	0.5	1.55
*E. coli* ^b^	–	–	–	–	–	–	0.5	1.55
*V. vulnificus* ^b^	–	–	–	–	–	–	2	6.19
*P. aeruginosa* ^b^	–	–	–	–	–	–	2	6.19
*M. luteus* ^b^	–	–	–	–	–	–	0.5	1.55
*C. diplodiella* ^c^	8	24.10	4	12.58	2	6.06	0.5	1.55
*P. digitatum* ^c^	8	24.10	8	25.16	8	24.24	0.5	1.55
*A. brassicae* ^c^	16	48.19	16	50.31	8	24.24	0.5	1.55
*C. spicifera* ^c^	4	12.05	4	12.58	4	12.12	0.5	1.55
*F. proliferatum* ^c^	2	6.02	4	12.58	4	12.12	0.5	1.55
*C. gloeosporioides* ^c^	4	12.05	4	12.58	16	48.48	1	3.10

^a^“–”: MIC > 64 μg/mL. ^b^Chloramphenicol as positive control. ^c^Amphotericin B as positive control.

## Conclusion

In conclusion, two new ovalicin sesquiterpenoid derivatives with a thioether bond, namely, pseudallenes A (**1**) and B (**2**), together with three known analogues **3**–**5**, were isolated and identified from the cold seep-derived fungus *P. boydii* CS-793. To date, only three sulfur-containing ovalicin sesquiterpenoids have been reported [10,12]. Moreover, the first characterized crystal structure of an ovalicin-type sesquiterpenoid was obtained, which further confirmed the structures and absolute configurations of compounds **1**–**3**. Biological evaluation revealed that compounds **1**–**3** exhibit potent antifungal activities against the plant pathogenic fungi *C. diplodiella*, *P. digitatum*, *A. brassicae*, *C. spicifera*, *F. proliferatum*, and *C. gloeosporioides,* with MIC values ranging from 2 to 16 μg/mL.

## Experimental

**General experimental procedures.** Melting points were determined by an SGW X-4 micro-melting point apparatus. Optical rotations were measured in MeOH using an Optical Activity AA-55 polarimeter. UV spectra were obtained with a PuXi TU-1810 UV–visible spectrophotometer. NMR spectra data were recorded on a Bruker Avance 500 or 600 MHz spectrometer using solvent chemical shifts (DMSO: δ_H_/δ_C_ 2.50/39.52) as reference. HRESIMS data were measured using an API QSTAR Pulsar 1 mass spectrometer. HPLC was performed on a Dionex HPLC system equipped with a P680 pump, an ASI-100 automated sample injector, and a UVD340U multiple-wavelength detector controlled by Chromeleon software (version 6.80). LC–MS were obtained with a Bruker maXis plus Q-TOF. Column chromatography was carried out using commercially available silica gel (200–300 mesh, Qingdao Haiyang Chemical Co.), Lobar LiChroprep RP-18 (40–63 μm, Merck), and Sephadex LH-20 (Pharmacia). Thin-layer chromatography (TLC) was performed with precoated Si gel GF_254_ plates (Merck, Darmstadt, Germany). Solvents used for extraction and purification were distilled prior to use. Peptone from yeast extract was purchased from Sigma-Aldrich. Rice, monosodium glutamate, and corn steep liquor were purchased from China Oil & Foodstuffs Corporation.

**Fungal material.** The fungus *Pseudallescheria boydii* CS-793 was isolated from the cold seep sediment at the Northeast of the South China Sea, collected in September 2020. The fungal strain was identified as *P. boydii* according to the ITS (internal transcript spacer) region sequence, which is the same (98.98%) as that of *P. boydii* (accession no. OW986361). The sequence data of CS-793 have been deposited in GenBank with the accession no. OQ390095. The strain is preserved at the Key Laboratory of Experimental Marine Biology, Institute of Oceanology, Chinese Academy of Sciences (IOCAS).

**Fermentation**, **extraction, and isolation.** Fermentation and extraction were performed in a manner analogous to reference [6]. For chemical investigations, rice solid medium containing rice (100 g/ﬂask), peptone from animal (0.3 g/ﬂask), yeast extract (0.5 g/ﬂask), corn steep liquor (0.2 g/ﬂask), monosodium glutamate (0.1 g/ﬂask), and naturally sourced and ﬁltered seawater (acquired from the Huiquan Gulf of the Yellow Sea near the campus of IOCAS, 100 mL/ﬂask) were autoclaved at 120 °C for 20 min before inoculation. The fresh mycelia of the fungus *P. boydii* CS-793 were incubated in a shaker on PDB medium at 28 °C for five days, which were then inoculated into the noted rice solid medium in 1 L Erlenmeyer flasks and static cultivation for 30 days at room temperature. After fermentation, it was fragmented mechanically and extracted thoroughly with EtOAc. The combined extracts were filtered and concentrated under reduced pressure to give 123.6 g of an organic extract.

The EtOAc extract was subjected to Si gel VLC (vacuum liquid chromatography) and fractionated using solvent mixtures of increasing polarity consisting of petroleum ether (PE) and EtOAc 20:1 to 1:1 and finally with CH_2_Cl_2_/MeOH 20:1 to 1:1 to yield nine fractions (Frs. 1–9). Fr.4 (2.1 g) was further purified by reversed-phase column chromatography (CC) over Lobar LiChroprep RP-18 with a MeOH/H_2_O gradient (from 1:9 to 10:0) to afford ten subfractions (Fr. 4.1–Fr. 4.10). Compound **3** (58.7 mg, *t*_R_ = 12.0 min) was isolated by semipreparative HPLC (Elite ODSBP column, 5 μm; 10 × 250 mm; 75% MeOH/H_2_O, 3 mL/min) from Fr. 4.3 (5.8 g). Fr. 5 (6.5 g) was further fractionated by CC over Lobar LiChroprep RP-18 eluting with a MeOH/H_2_O gradient (from 1:9 to 10:0) to yield 10 subfractions (Frs. 5.1–5.10). Fr. 5.3 (258 mg) was further purified by CC on silica gel eluting with a CH_2_Cl_2_/MeOH gradient (from 200:1 to 100:1) and then by preparative TLC (plate: 20 × 20 cm, developing solvents: ether/acetone 2:1) to afford compound **4** (8.6 mg). Fr. 5.4 (538 mg) was separated by CC on Si gel and Sephadex LH-20 (MeOH), after that compounds **1** (12.5 mg, *t*_R_ = 14.0 min) and **5** (6.0 mg, *t*_R_ = 15.0 min) were isolated by semipreparative HPLC (Elite ODSBP column, 5 μm; 10 × 250 mm; 71% MeOH/H_2_O, 3 mL/min). Fr. 6 (10.5 g) was fractionated by CC over Lobar LiChroprep RP-18 eluting with a MeOH/H_2_O gradient (from 1:9 to 10:0) to yield 10 subfractions (Frs. 6.1–6.10). Then, compound **2** (13.7 mg) was isolated by CC on Si gel (CH_2_Cl_2_/MeOH, 250:1 to 50:1) and preparative TLC (plate: 20 × 20 cm, developing solvent: ether/acetone 2:1) from Fr. 6.3 (578 mg).

Pseudallene A (**1**): colorless crystals (MeOH); mp 115–117 °C; [α]_D_^25^ +20.0 (*c* 0.4, MeOH); ^1^H and ^13^C NMR data, see Table 2; HRESIMS (*m/z*): [M + H]^+^ calcd for C_16_H_29_O_5_S, 333.1730; found: 333.1733).

Pseudallene B (**2**): colorless crystals (MeOH); mp 171**–**175 °C; [α]_D_^25^ +53.3 (*c* 0.3, MeOH); ^1^H and ^13^C NMR data, see Table 2; HRESIMS (*m/z*): [M + H]^+^ calcd for C_15_H_27_O_5_S, 319.1573; found: 319.1568..

**X-ray crystallographic analysis of compounds 1**–**3** [16]. Compounds **1**–**3** were crystallized in MeOH. All crystallographic data were collected on a Bruker Smart-1000 or Bruker D8 Venture CCD diffractometer using Cu Kα radiation (λ = 1.54178 Å). The data were corrected for absorption by using the program SADABS [17]. The structures were solved by direct methods using the SHELXTL software package [18–19]. All non-hydrogen atoms were refined anisotropically. The H atoms were located by geometrical calculations, and their positions and thermal parameters were fixed during the structure refinement. The absolute structures were determined by refinement of the Flack parameters [20], based on anomalous scattering. The structures were optimized by full matrix least-squares techniques.

Crystal data for compound **1**: C_16_H_28_O_5_S, fw = 332.15; orthorhombic space group *P*2(1)2(1)2(1), unit cell dimensions *a* = 15.0176(3) Å, *b* = 24.2644(4) (11) Å, *c* = 29.3542(5) (4) Å, *V* = 4749.6 (3) Å^3^, α = β = γ = 90°, *Z* = 4, *d*_calcd_ = 1.237 mg/m^3^, crystal dimensions 0.18 × 0.16 × 0.12 mm, μ = 1.783 mm^−1^, *F*(000) = 4312. The 67427 measurements yielded 19396 independent reflections after equivalent data were averaged. The final refinement gave *R*_1_ = 0.0512 and w*R*_2_ = 0.1339 [*I* > 2*σ*(*I*)]. The absolute structure parameter was 0.019(6).

Crystal data for compound **2**: C_15_H_26_O_5_S, fw = 318.15, triclinic space group *P*1, unit cell dimensions *a* = 6.50160(10) Å, *b* = 7.7295(2) Å, *c* = 17.5958(4) Å, *V* = 794.00(3) Å^3^, α = 85.4270(10)°, β = 81.7990(10)°, γ = 65.1510(10)°, *Z* = 1, *d*_calcd_ = 1.332 mg/m^3^, crystal dimensions 0.18 × 0.15 × 0.12 mm, μ = 1.979 mm^−1^, *F*(000) = 344. The 18694 measurements yielded 5468 independent reflections after equivalent data were averaged. The final refinement gave *R*_1_ = 0.0287 and w*R*_2_ = 0.0714 [*I* > 2*σ*(*I*)]. The absolute structure parameter was 0.048(7).

Crystal data for compound **3**: C_16_H_26_O_5_S, fw = 330.43, triclinic space group *P*6(1), unit cell dimensions *a* = 13.99630(10) Å, *b* = 13.99630(10) Å, *c* = 30.1665(6) Å, *V* = 5117.78(13) Å^3^, α = 90°, β = 90°, γ = 120°, *Z* = 12, *d*_calcd_ = 1.287 mg/m^3^, crystal dimensions 0.16 × 0.14 × 0.12 mm, μ = 1.863 mm^−1^, *F*(000) = 2136. The 65189 measurements yielded 6250 independent reflections after equivalent data were averaged. The final refinement gave *R*_1_ = 0.0317 and w*R*_2_ = 0.0848 [*I* > 2*σ*(*I*)]. The absolute structure parameter was 0.024(6).

**Antibacterial assay.** The antibacterial activities against human pathogenic bacteria (*Escherichia coli* QDIO-1 and *Pseudomonas aeruginosa* QDIO-4) and aquatic pathogens (*Edwardsiella tarda* QDIO-2, *Micrococcus luteus* QDIO-3, *V. anguillarum* QDIO-6, *Vibrio harveyi* QDIO-7, and *V. vulnificus* QDIO-10), as well as plant pathogenic fungi (*Colletotrichum gloeosporioides* QDAU-2, *Alternaria brassicae* QDAU-11, *Penicillium digitatum* QDAU-14, *Coniothyrium diplodiella* QDAU-15, *Curvularia spicifera* QDAU-18, and *Fusarium proliferatum* QDAU-19) were determined by a serial dilution technique using 96-well microtiter plates [21] with minor modifications as our previously report [22]. Briefly, the bacteria were cultivated in the LB broth medium at 37 °C for the human pathogenic bacteria, while the temperature was 28 °C for the aquatic pathogens, and they were prepared at a concentration of 1.5 × 10^8^ CFU/mL. Tested compounds and positive control (chloramphenicol) were dissolved in DMSO to give a stock solution with DMSO as a negative control. Then, 95 μL of thus prepared bacteria and 5 μL of the above tested compounds or chloramphenicol (with the final concentration of 0.5, 1, 2, 4, 8, 16, 32, 64 μg/mL) were added into the 96-well plates and cultivated at 37 °C or 28 °C for 24 h. Optical density at 600 nm was read by a multi-detection microplate reader (Infinite M1000 Pro, Tecan). The human pathogenic bacteria and aquatic pathogenic strains were offered by the Institute of Oceanology, Chinese Academy of Sciences.

## Supporting Information

File 1 Selected 1D and 2D NMR, and HRESIMS spectra of compounds **1** and **2**, and 1D NMR spectra of compounds **3**–**5**.

File 2 X-ray crystallographic files of compounds **1–3**.

## Data Availability

All data that supports the findings of this study is available in the published article and/or the supporting information to this article.
