# Supplementary material for: Pseudallenes A and B, new sulfur-containing ovalicin sesquiterpenoid derivatives with antimicrobial activity from the deep-sea cold seep sediment-derived fungus Pseudallescheria boydii CS-793
Source: Beilstein J Org Chem. 2024 Feb 28;20:470–8. doi: 10.3762/bjoc.20.42 (PMC10910587; doi:10.3762/bjoc.20.42)
Supplement: File 1 — Selected 1D and 2D NMR, and HRESIMS spectra of compounds 1 and 2, and 1D NMR spectra of compounds 3–5. [file Beilstein_J_Org_Chem-20-470-s001.pdf]

BEILSTEIN JOURNAL OF ORGANIC CHEMISTRY

Supporting Information

for

Pseudallenes A and B, new sulfur-containing ovalicin sesquiterpenoid derivatives with antimicrobial activity from the deep-sea cold seep sediment-derived fungus *Pseudallescheria boydii* CS-793

Zhen Ying, Xiao-Ming Li, Sui-Qun Yang, Hong-Lei Li, Xin Li, Bin-Gui Wang and Ling-Hong Meng

Beilstein J. Org. Chem. **2024,** *20,* 470–478. doi:10.3762/bjoc.20.42

Selected 1D and 2D NMR, and HRESIMS spectra of compounds 1 and 2, and 1D NMR spectra of compounds 3–5

License and Terms: This is a supporting information file under the terms of the Creative Commons Attribution License (https://creativecommons.org/licenses/by/4.0). Please note that the reuse, redistribution and reproduction in particular requires that the author(s) and source are credited and that individual graphics may be subject to special legal provisions.
The license is subject to the *Beilstein Journal of Organic Chemistry* terms and conditions: (https://www.beilstein-journals.org/bjoc/terms)

Figure S1. ^{1}H NMR (500 MHz, DMSO-d_6) spectrum of compound **1**;
Figure S2. ^{13}C NMR (125 MHz, DMSO-d_6) and DEPT spectra of compound **1**;
Figure S3. COSY spectrum of compound **1**;
Figure S4. HSQC spectrum of compound **1**;
Figure S5. HMBC spectrum of compound **1**;
Figure S6. NOESY spectrum of compound **1**;
Figure S7. LC-MS spectrum of compound **1**;
Figure S8. ^{1}H NMR (500 MHz, DMSO-d_6) spectrum of compound **2**;
Figure S9. ^{13}C NMR (125 MHz, DMSO-d_6) and DEPT spectra of compound **2**;
Figure S10. COSY spectrum of compound **2**;
Figure S11. HSQC spectrum of compound **2**;
Figure S12. HMBC spectrum of compound **2**;
Figure S13. NOESY spectrum of compound **2**;
Figure S14. LC-MS spectrum of compound **2**;
Figure S15 ^{1}H NMR (500 MHz, $CDCl_3$) spectrum of compound **3**;
Figure S16. ^{13}C NMR (125 MHz, $CDCl_3$) and DEPT spectra of compound **3**;
Figure S17. COSY spectrum of compound **3**;
Figure S18. HSQC spectrum of compound **3**;
Figure S19. HMBC spectrum of compound **3**;
Figure S20. ^{1}H NMR (500 MHz, DMSO-d_6) spectrum of compound **4**;
Figure S21. ^{13}C NMR (125 MHz, DMSO-d_6) and DEPT spectra of compound **4**.
Figure S22. ^{1}H NMR (500 MHz, DMSO-d_6) spectrum of compound **5**;
Figure S23. ^{13}C NMR (125 MHz, DMSO-d_6) and DEPT spectra of compound **5**.
Table S1. Crystal data and structure refinement for compounds **1−3.**
Table S2. ^{1}H, ^{13}C, COSY, HMBC Spectroscopic Data for Compound **1** in DMSO-d_6.
Table S3. ^{1}H, ^{13}C, COSY, HMBC Spectroscopic Data for Compound **2** in DMSO-d_6.
Table S4. ^{1}H, ^{13}C, COSY, HMBC Spectroscopic Data for Compound **3** in $CDCl_3$.

Figure S1. ^{1}H NMR (500 MHz, DMSO-d_6) spectrum of compound **1**;

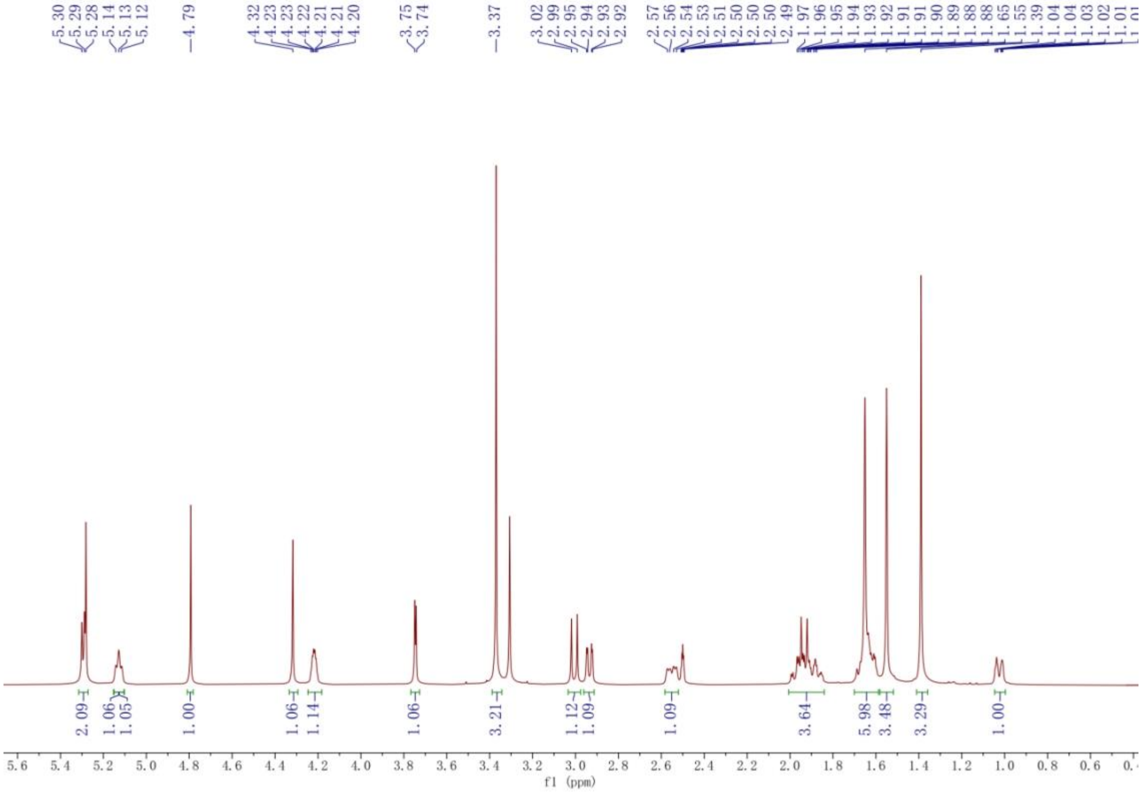

Figure S2. ^{13}C NMR (125 MHz, DMSO-d_6) and DEPT spectra of compound **1**;

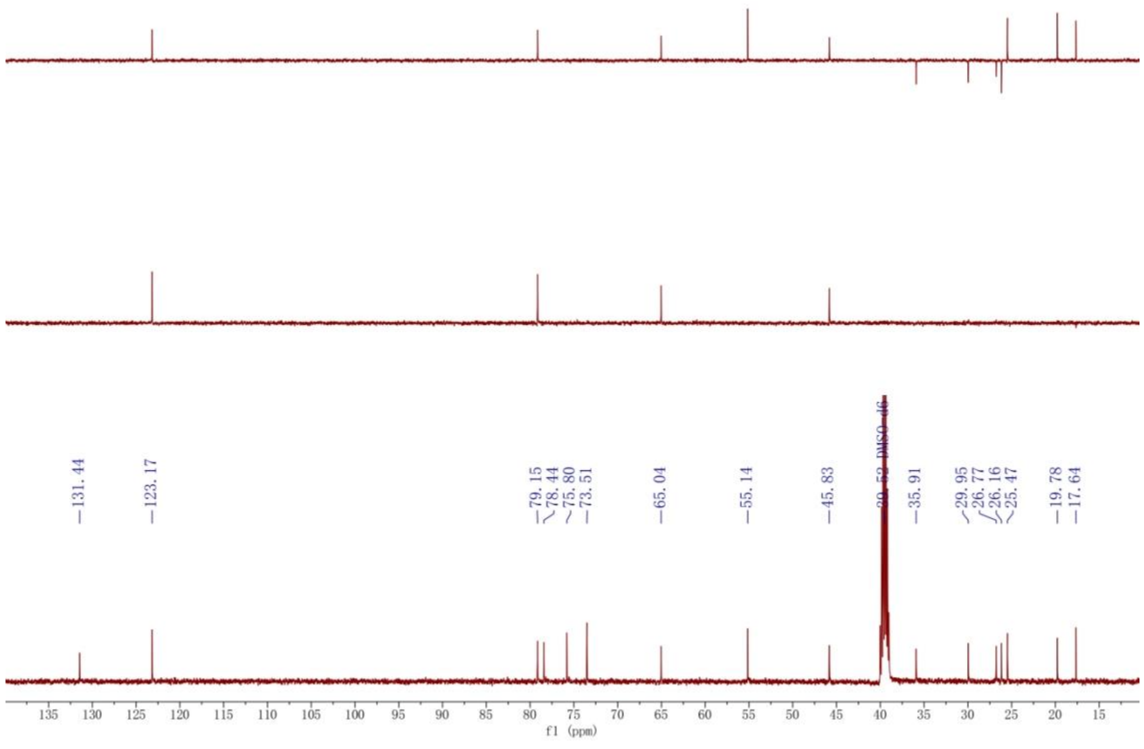

Figure S3. COSY spectrum of compound **1**;.

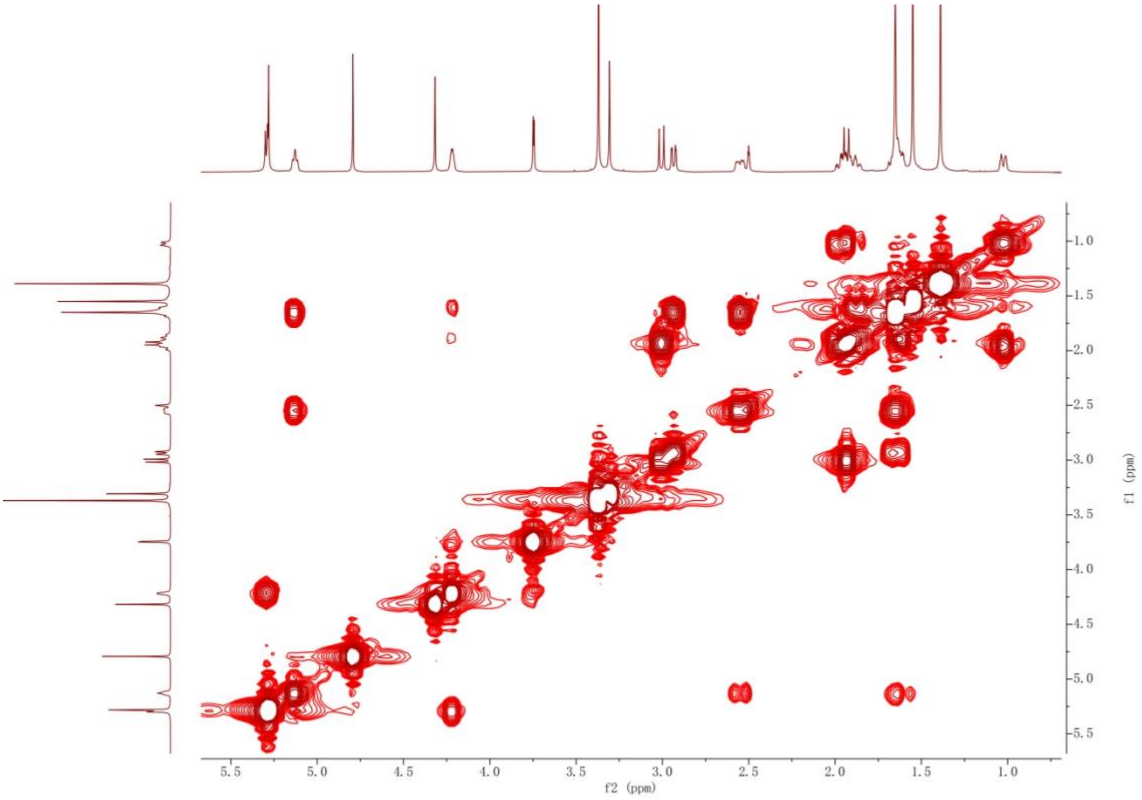

Figure S4. HSQC spectrum of compound **1**;

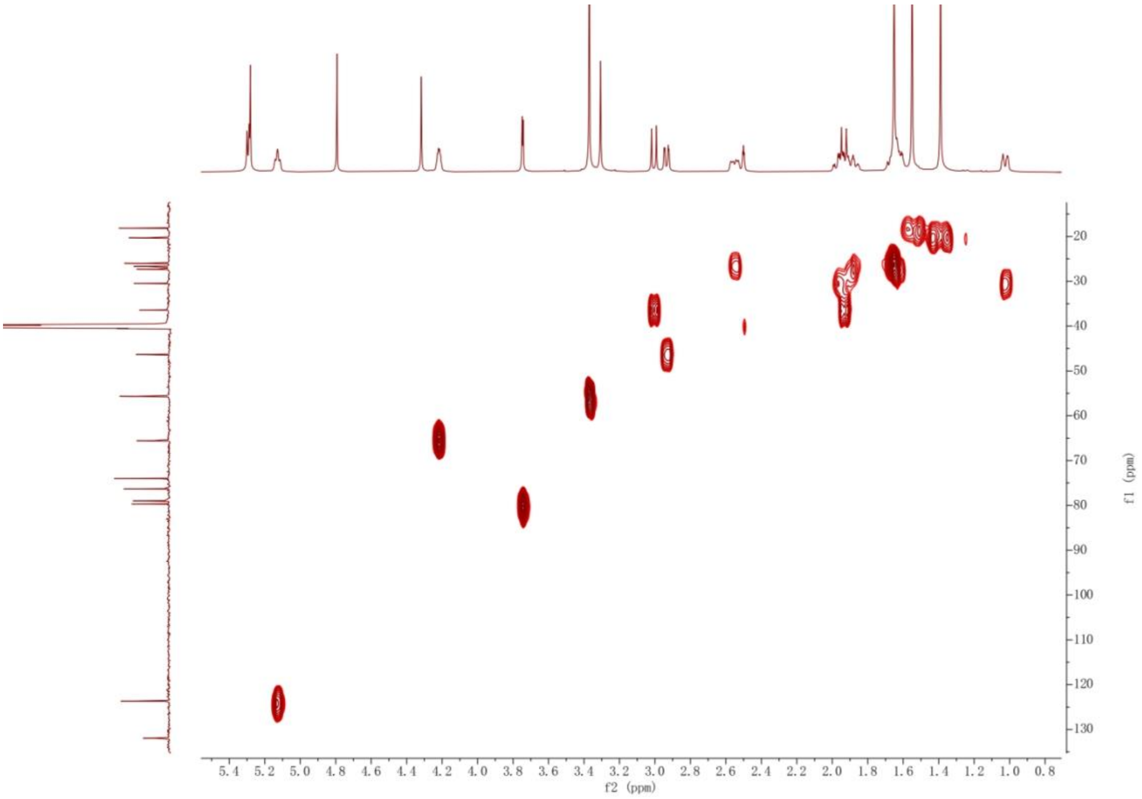

Figure S5. HMBC spectrum of compound **1**;

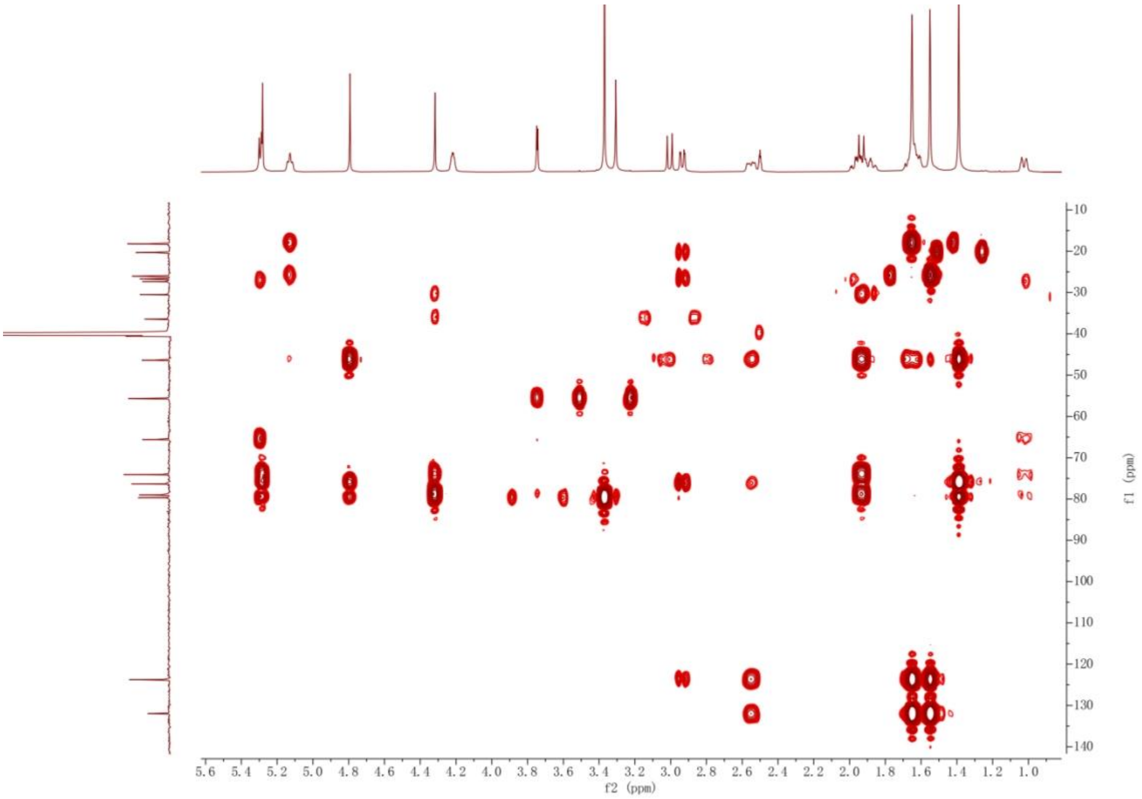

Figure S6. NOESY spectrum of compound **1**;

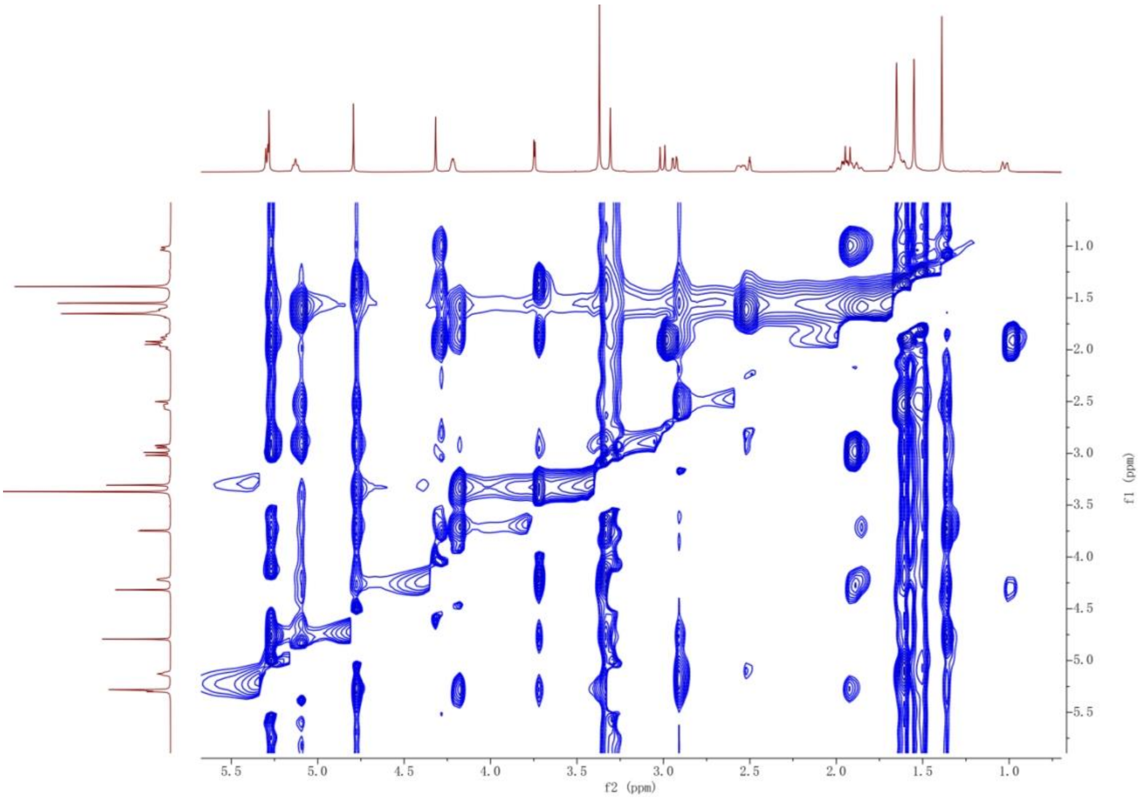

Figure S7. LC-MS spectrum of compound **1**;

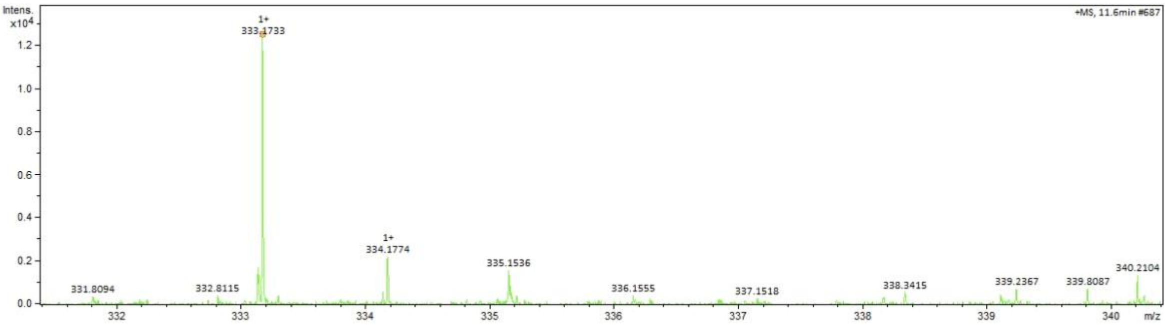

Figure S8. ^{1}H NMR (500 MHz, DMSO-d_6) spectrum of compound **2**;

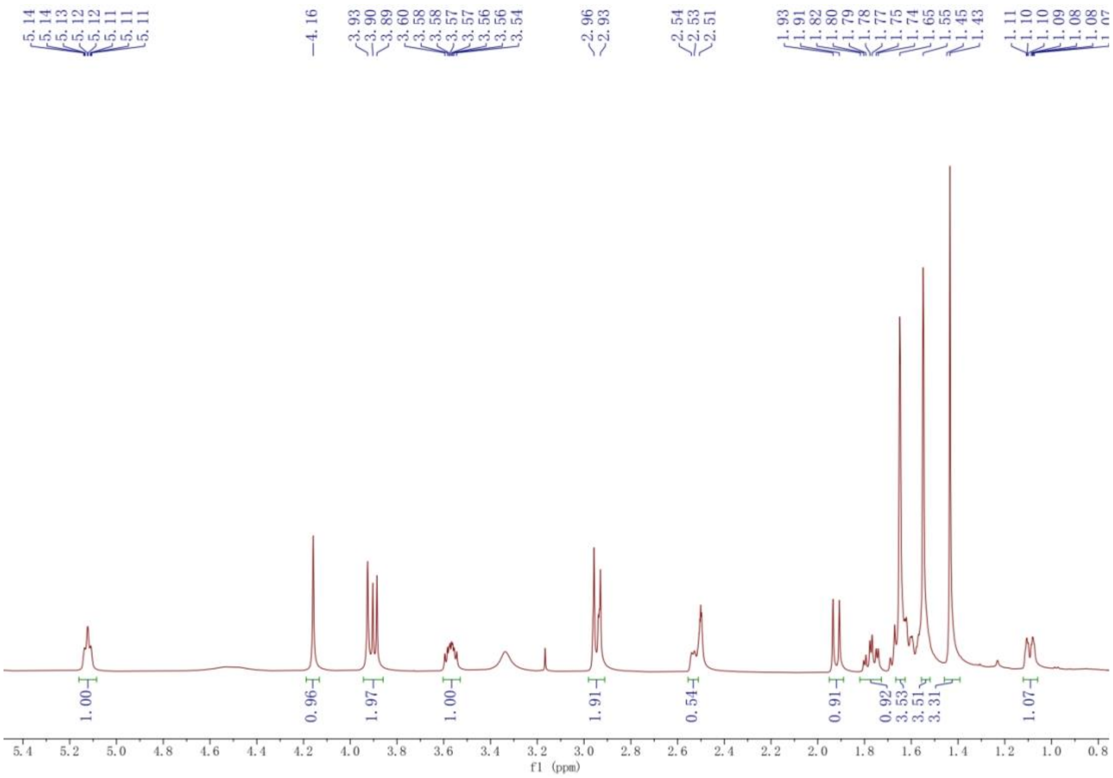

Figure S9. ^{13}C NMR (125 MHz, DMSO-d_6) and DEPT spectra of compound **2**;

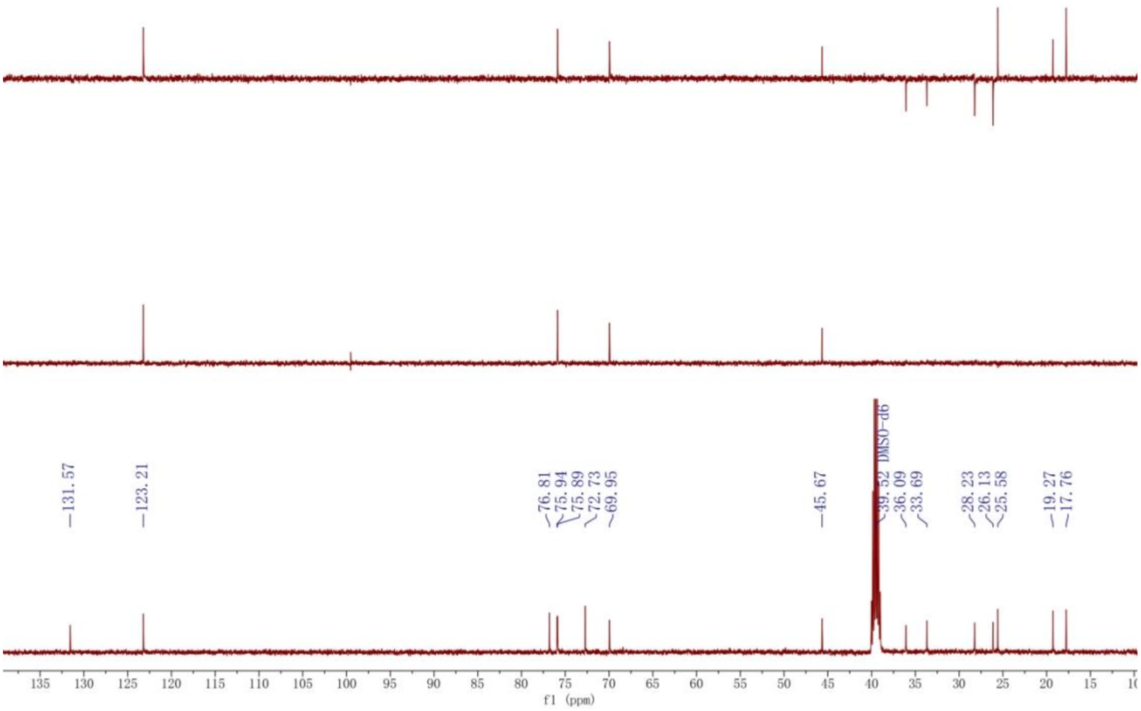

Figure S10. COSY spectrum of compound **2**;.

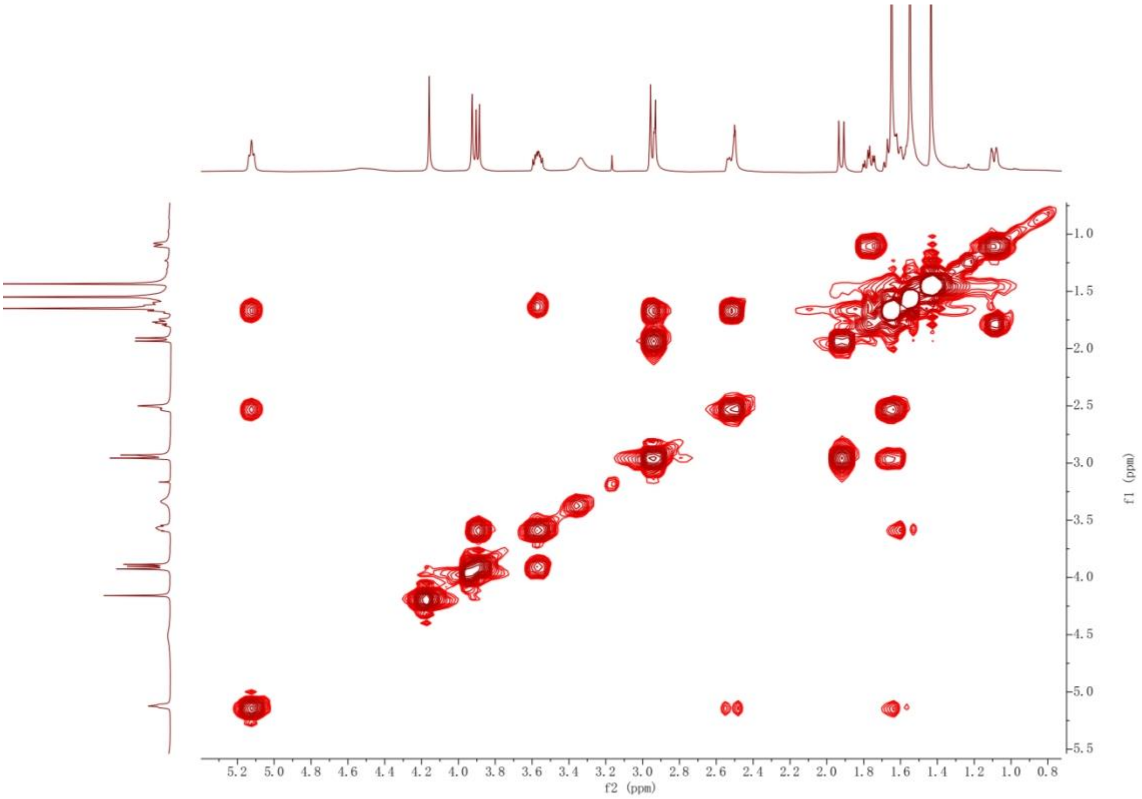

Figure S11. HSQC spectrum of compound **2**;

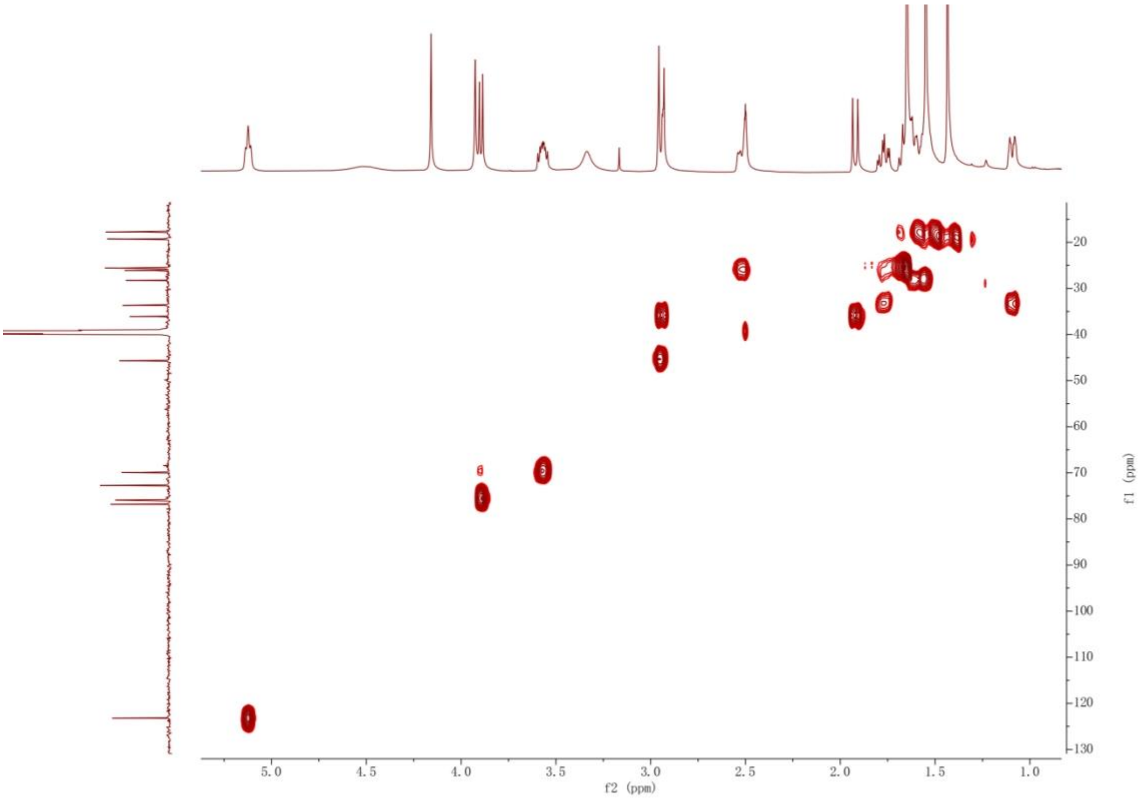

Figure S12. HMBC spectrum of compound **2**;

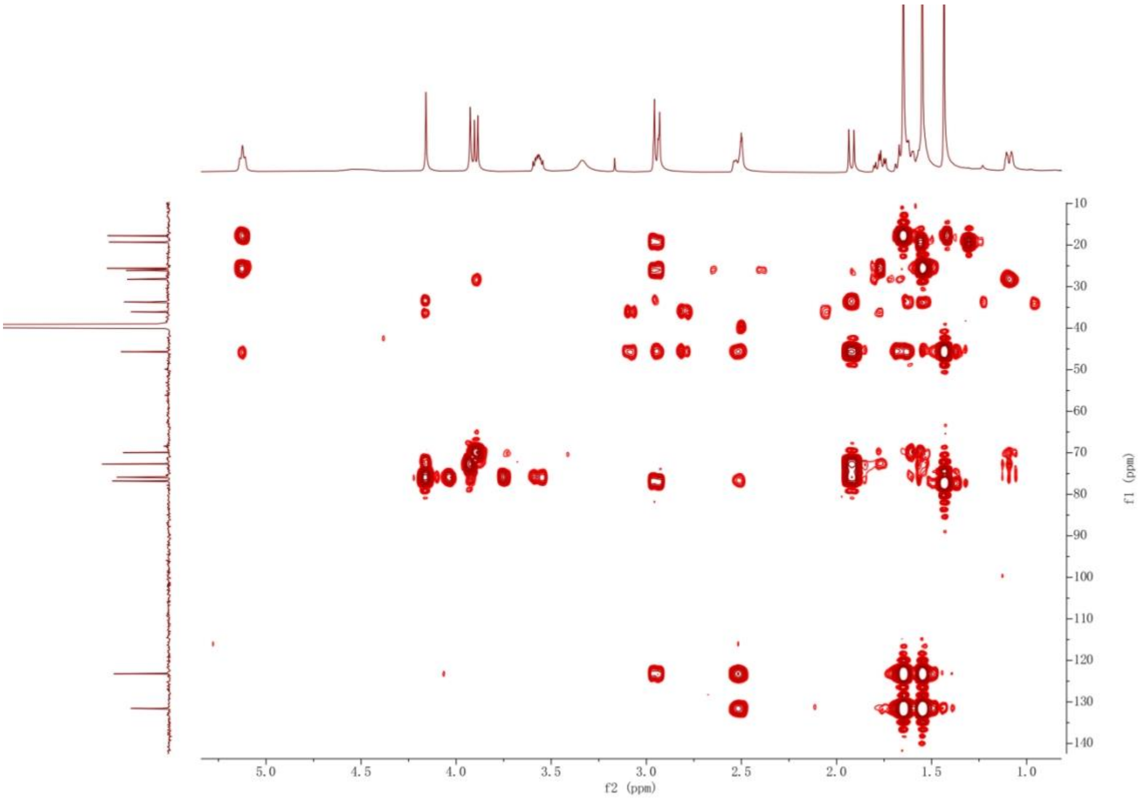

Figure S13. NOESY spectrum of compound **2**;

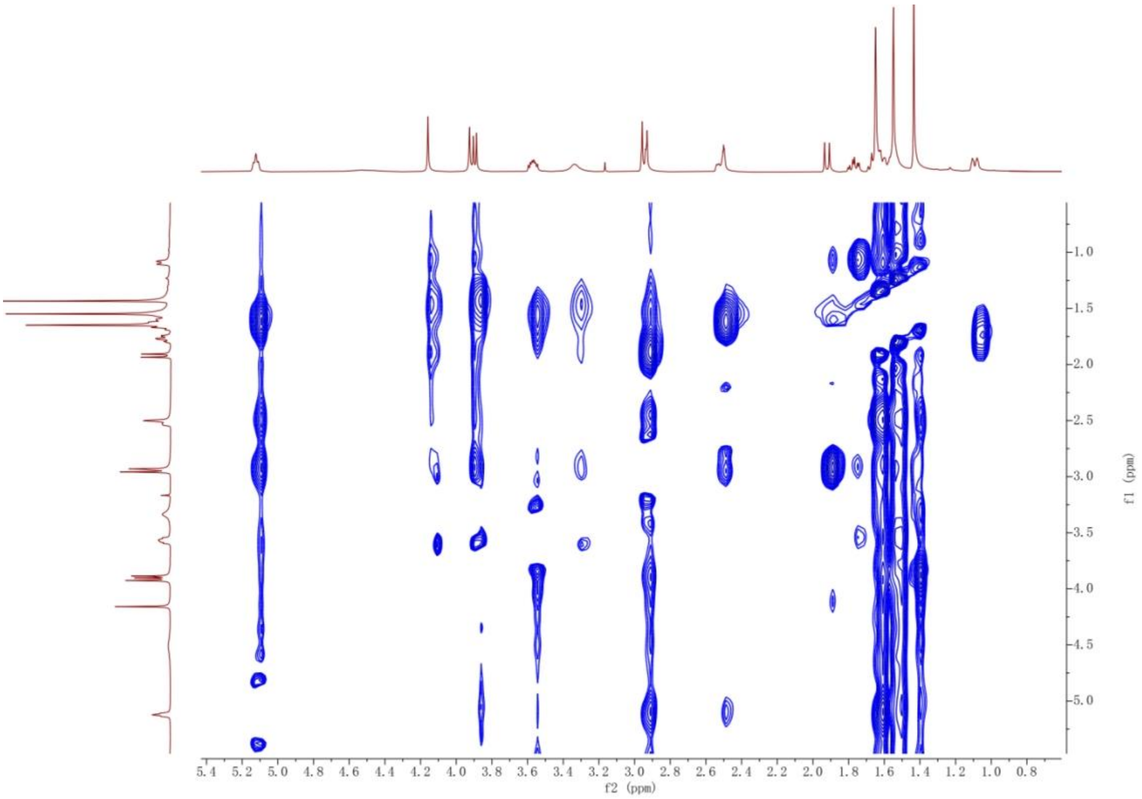

Figure S14. LC-MS spectrum of compound **2**;

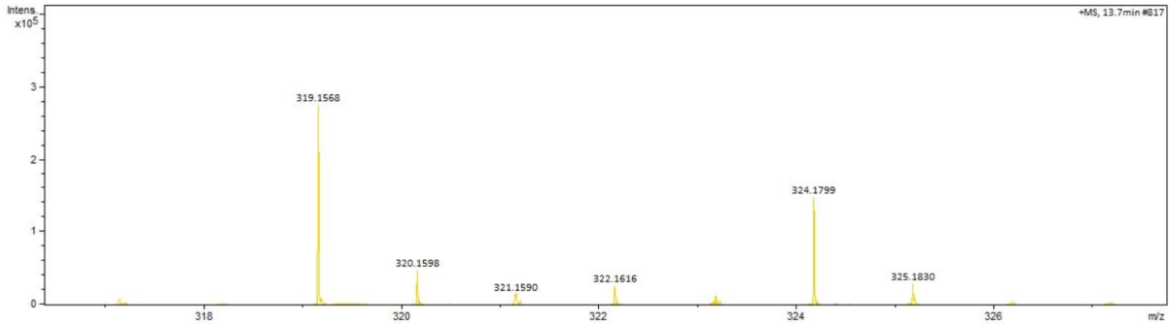

Figure S15. ^{1}H NMR (500 MHz, $CDCl_3$) spectrum of compound **3**;

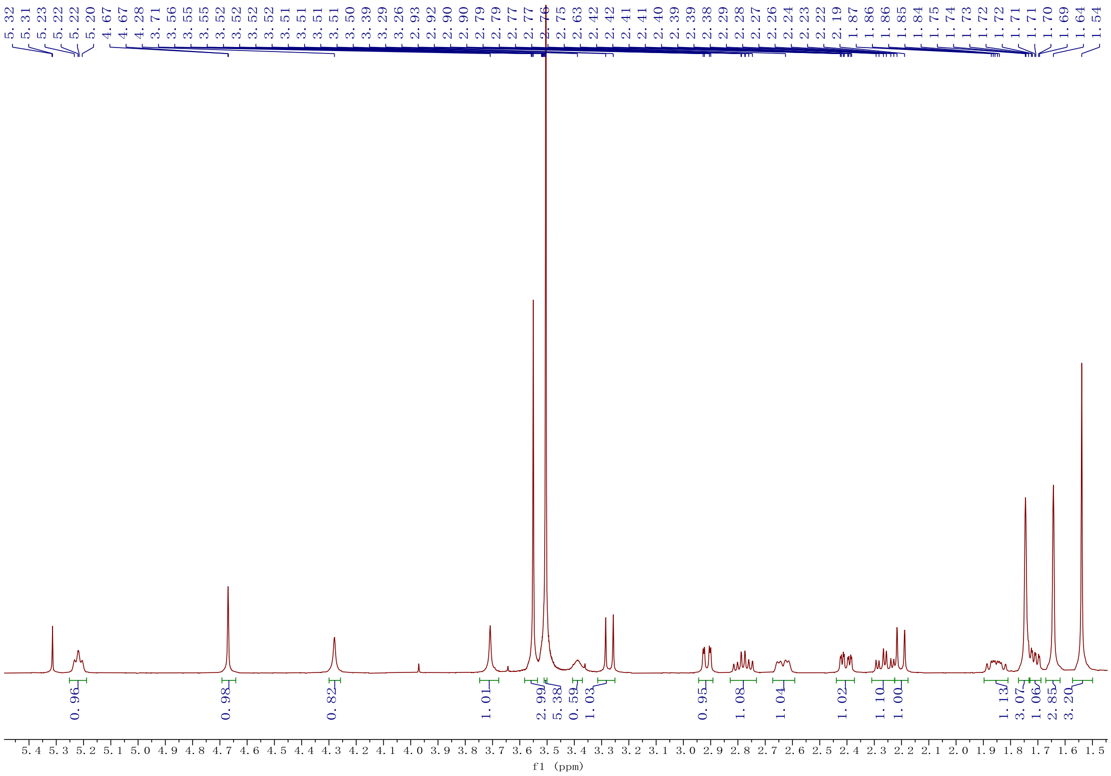

Figure S16. ^{13}C NMR (125 MHz, $CDCl_3$) and DEPT spectra of compound **3**;

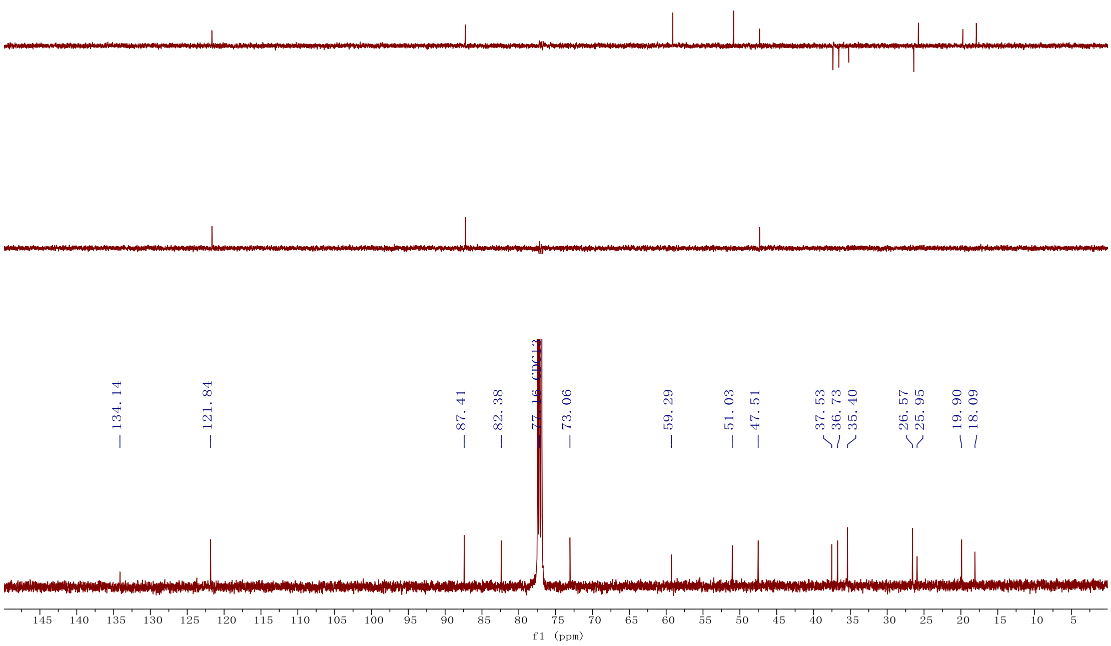

Figure S17. COSY spectrum of compound **3**;

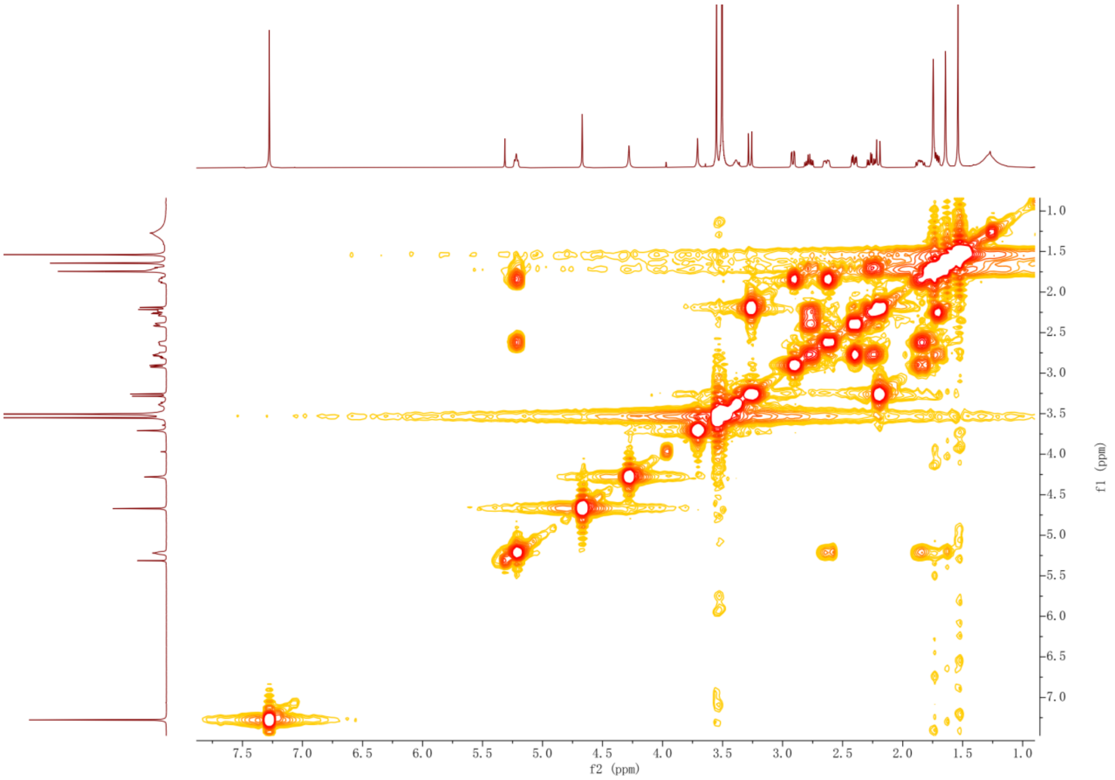

Figure S18. HSQC spectrum of compound **3**;

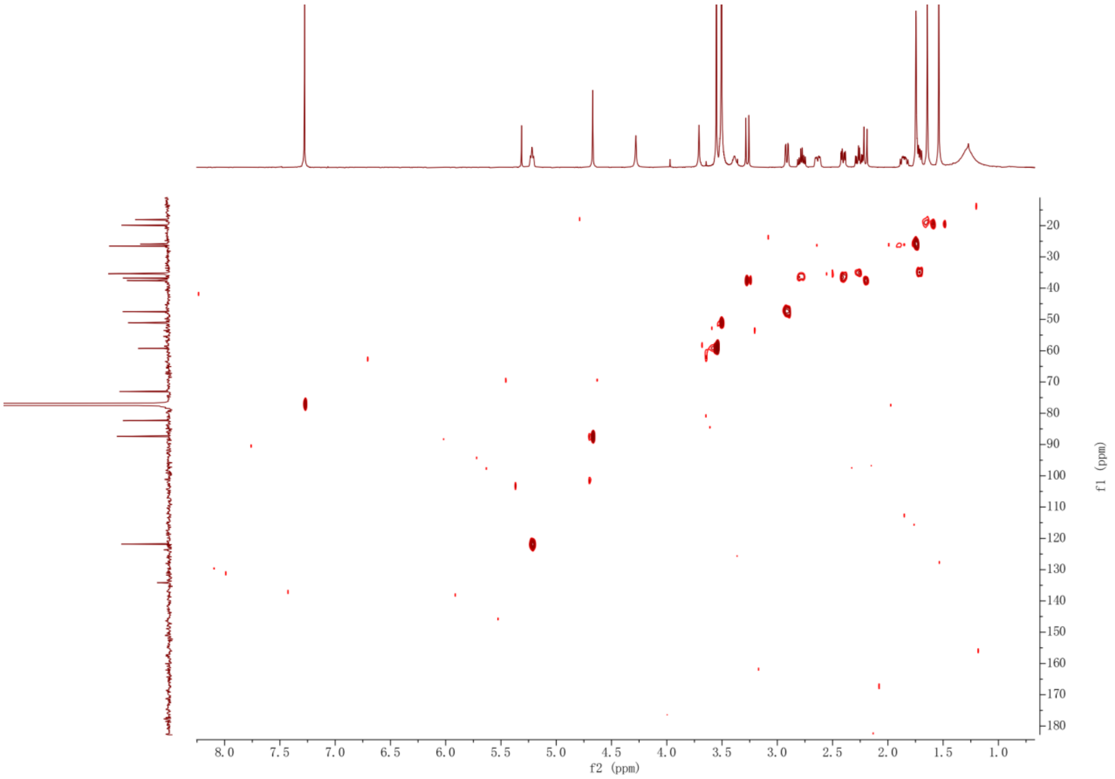

Figure S19. HMBC spectrum of compound **3**;

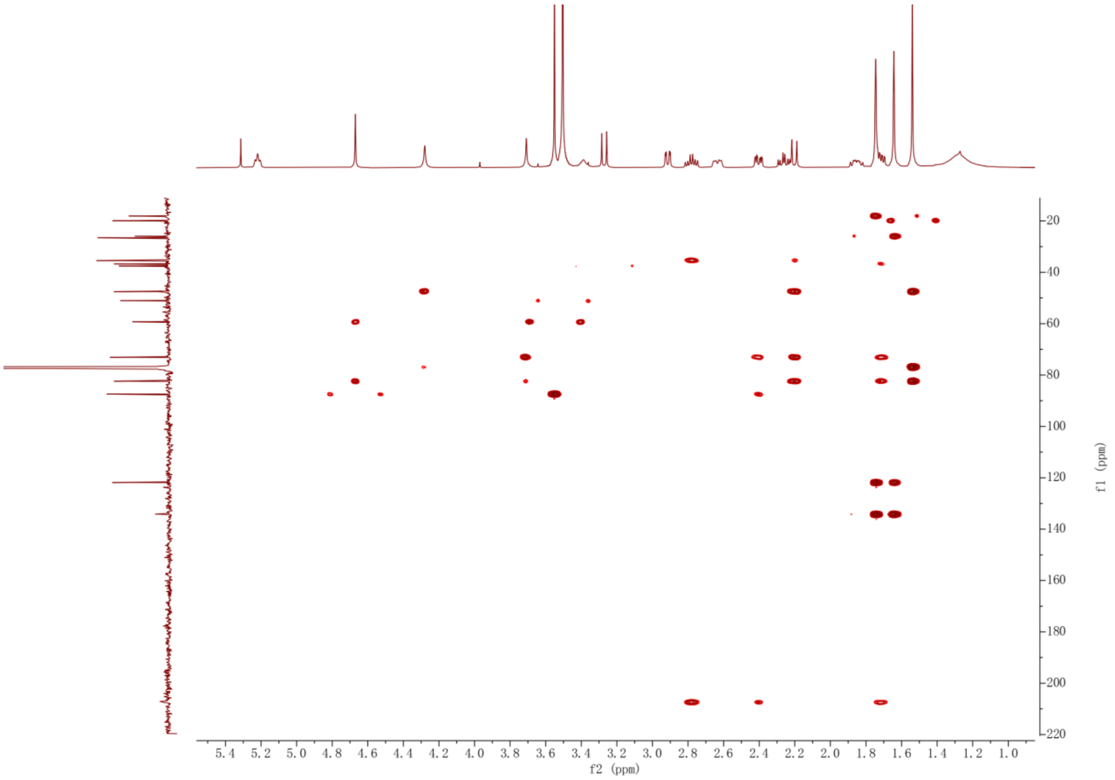

Figure S20. ^{1}H NMR (500 MHz, DMSO-d_6) spectrum of compound **4**;

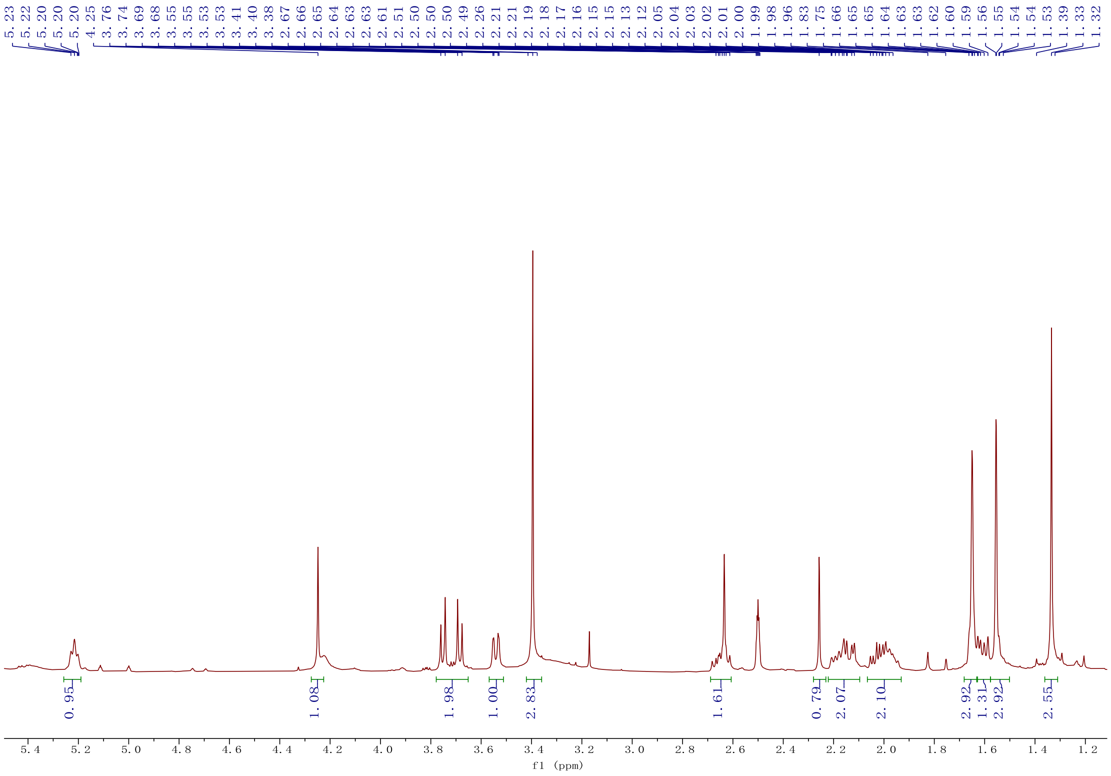

Figure S21. ^{13}C NMR (125 MHz, DMSO-d_6) and DEPT spectra of compound **4**;

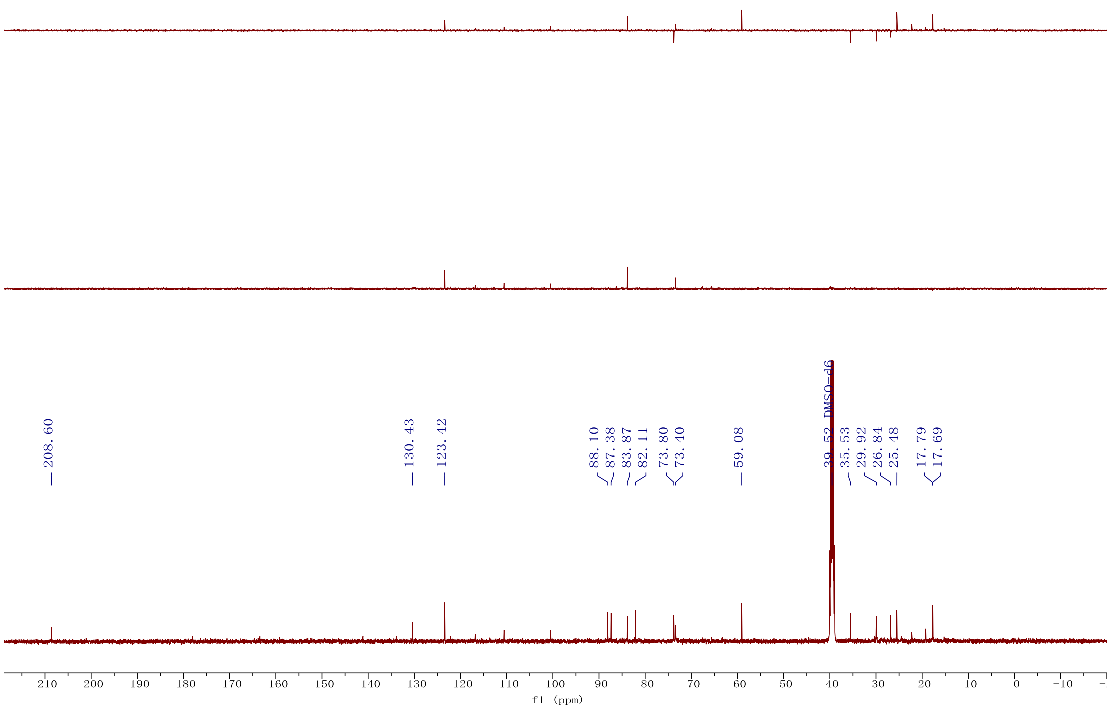

Figure S22. ^{1}H NMR (500 MHz, DMSO-d_6) spectrum of compound **5**;

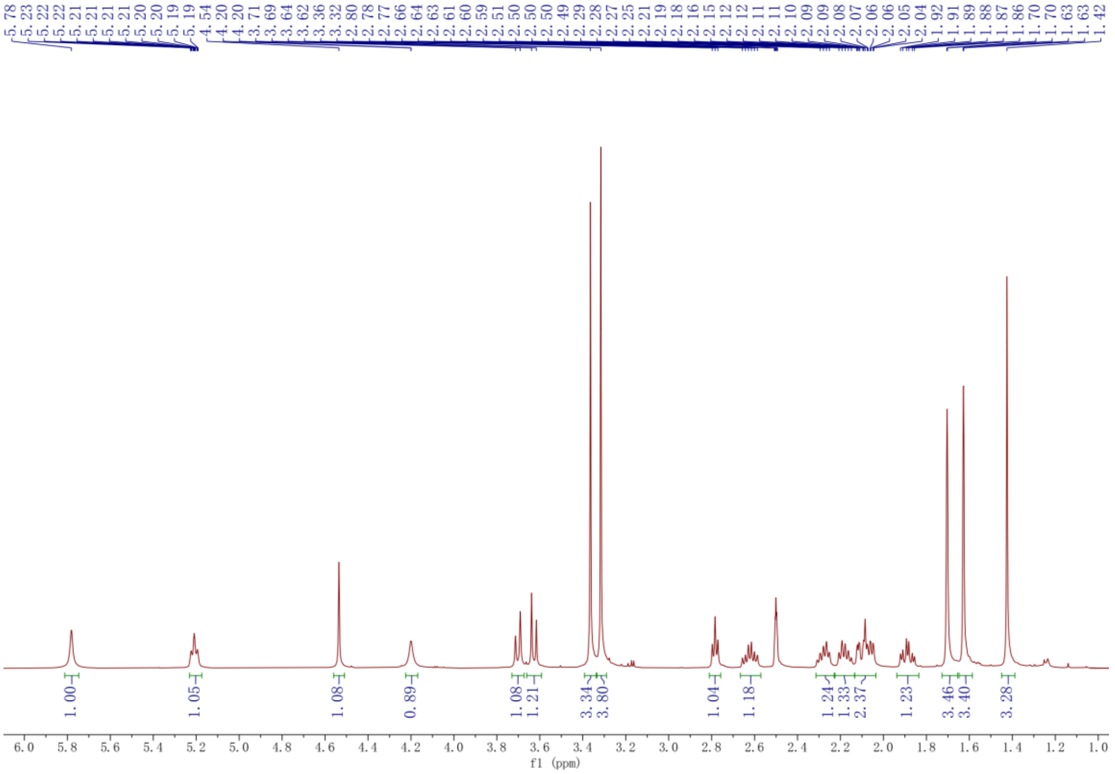

Figure S23. ^{13}C NMR (125 MHz, DMSO-d_6) and DEPT spectra of compound **5**.

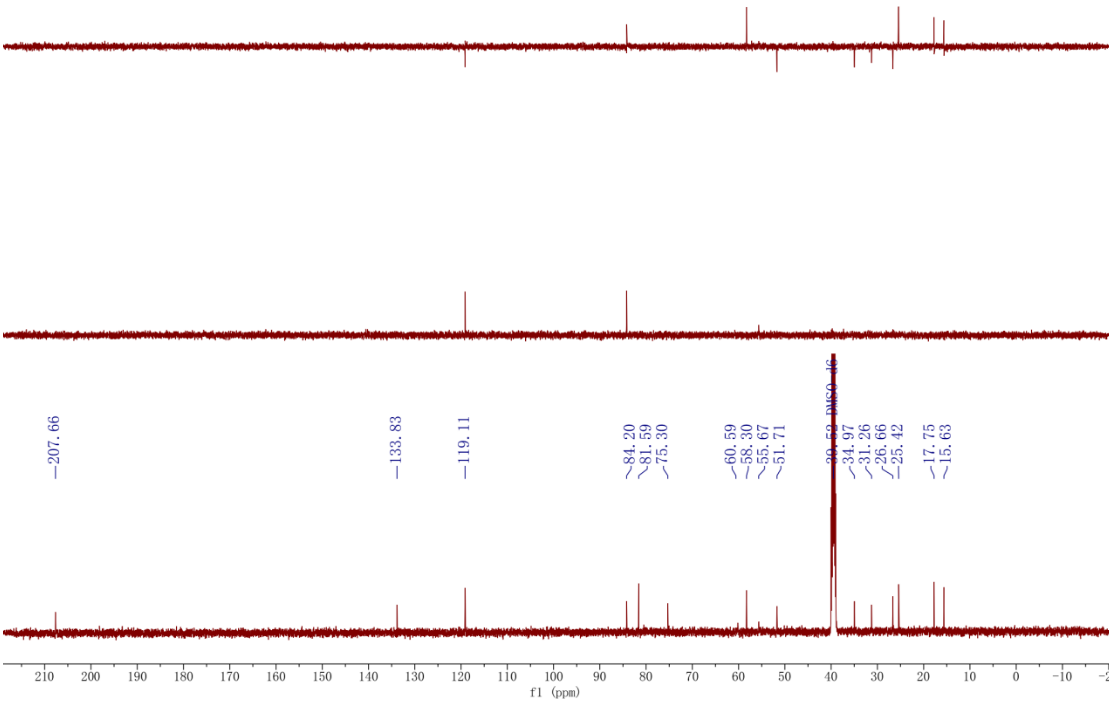

Table S1. Crystal data and structure refinement for compounds **1–3**.

Identification code	Compound **1**	Compound **2**	Compound **3**
Empirical formula	$C_{96} H_{166} O_{30} S_6$	$C_{30} H_{52} O_{10} S_2$	$C_{16} H_{26} O_5 S$
Formular weight	1992.64	636.83	330.43
Temperature	173(2) K	173(2) K	173(2) K
Wavelength	1.54178 A	1.54178 A	1.54178 A
Crytal system, space group	Orthorhombic, P2(1)2(1)2(1)	Triclinic, P1	Hexagonal, P6(1)
Unit cell dimensions	a = 15.0176(3) A<br>alpha = 90 deg.<br>b = 24.2644(4) A<br>beta = 90 deg.<br>c = 29.3542(5) A<br>gamma = 90 deg.	a = 6.50160(10) A<br>alpha = 85.4270(10) deg.<br>b = 7.7295(2) A<br>beta = 81.7990(10) deg.<br>c = 17.5958(4) A<br>gamma = 65.1510(10) deg.	a = 13.99630(10) A<br>alpha = 90 deg.<br>b = 13.99630(10) A<br>beta = 90 deg<br>c = 30.1665(6) A<br>gamma = 120 deg.
Volume	10696.5(3) A^3	794.00(3) A^3	5117.78(13) A^3
Z, Calculated density	4, 1.237 Mg/m^3	1, 1.332 Mg/m^3	12, 1.287 Mg/m^3
Absorption coeffcient	1.783 mm^-1	1.979 mm^-1	1.863 mm^-1
F(000)	4312	344	2136
Crystal size	0.180 x 0.160 x 0.120 mm	0.180 x 0.150 x 0.120 mm	0.160 x 0.140 x 0.120 mm
Theta range for data collection	2.362 to 68.182 deg.	2.538 to 66.525 deg.	3.646 to 68.266 deg
Limiting indices	-17<=h<=17, -21<=k<=29, -33<=l<=35	-7<=h<=7, -9<=k<=9, -20<=l<=20	-16<=h<=16, -16<=k<=16, -36<=l<=36
Reflections collected/unique	67427 / 19396 [R(int) = 0.0550]	18694 / 5468 [R(int) = 0.0381]	65189 / 6250 [R(int) = 0.0627]
Data/restraints/ parameters	19396 / 0 / 1252	5468 / 3 / 415	6250 / 1 / 442
Goodness-of-fit on F^2	1.000	1.033	0.947
Final R indices [I>2sigma(I)]	R1 = 0.0512, wR2 = 0.1339	R1 = 0.0287, wR2 = 0.0714	R1 = 0.0317, wR2 = 0.0848

R indices (all data)	R1 = 0.0605, wR2 = 0.1430	R1 = 0.0322, wR2 = 0.0736	R1 = 0.0361, wR2 = 0.0883
Absolute structure parameter	0.019(6)	0.048(7)	0.024(6)
Extinction coefficient	n/a	n/a	n/a
Largest diff. Peak and hole	0.792 and -0.378 e.A^-3	0.344 and -0.160 e.A^-3	0.175 and -0.189 e.A^-3

Table S2. ^{1}H, ^{13}C, COSY, HMBC Spectroscopic Data for Compound **1** in DMSO-d_6.

no.	**1**		**COSY**	**HMBC**
	δ_C[a]	δ_H[b], mult (*J* in Hz)		
1	78.4, C			
2	79.2, CH	3.75 d (3.1)	H-3	C-8
3	65.0, CH	4.22 dq (6.1, 3.1)	H-2, H-4, 3-OH	
4	26.8, CH_2	α 1.61 overlap	H-3, H-5	C-2, C-6
		β 1.87 overlap		
5	30.0, CH_2	α 1.03 dt (11.6, 2.7)	H-4	C-1, C-3, C-6
		β 1.97 overlap		
6	73.5, C			
7	75.8, C			
8	45.8, CH	2.93 dd (11.5, 2.4)	H-9	C-10
9	26.2, CH_2	α 1.68 overlap	H-8, H-10	C-11
		β 2.55 dd (15.2, 6.3)		
10	123.2, CH	5.14 t (6.9)	H-9	C-12, C-13
11	131.4, C			
12	25.5, CH_3	1.65 s		C-10, C-13
13	17.6, CH_3	1.55 s		C-10, C-12
14	35.9, CH_2	α 1.93 d (13.7)		C-1, C-5, C-6, C-8
		β 3.01 d (13.7)		
15	19.8, CH_3	1.39 s		C-1, C-7, C-8
1-OH		5.28 s		C-2, C-6
2-OCH_3/OH	55.1, CH_3	3.37 s		C-2
3-OH		5.29 d (6.0)		C-2, C-4
6-OH		4.32 s		C-5, C-6, C-14
7-OH		4.79 s		C-1, C-7, C-8

Table S3. ^{1}H, ^{13}C, COSY, HMBC Spectroscopic Data for Compound **2** in DMSO-d_6.

no.	**2**		**COSY**	**HMBC**
	δ_C[a]	δ_H[b], mult (J in Hz)		
1	76.8, C			
2	75.9, CH	3.89 d (8.7)	H-3	C-4
3	70.0, CH	3.57 ddd (11.1, 8.7, 5.5)	H-2, H-4,	C-1
4	28.2, CH_2	α 1.56 overlap	H-3, H-5	C-6
		β 1.60 overlap		
5	33.7, CH_2	α 1.09 ddd (13.2, 4.3, 2.7)	H-4	C-3, C-6
		β 1.77 td (13.2, 4.8)		
6	72.7, C			
7	76.8, C			
8	45.7, CH	2.94 overlap	H-9	C-7
9	26.1, CH_2	α 1.67 overlap	H-8, H-10	C-11
		β 2.53 overlap		
10	123.2, CH	5.12 t (6.4)	H-9	C-8, C-12, C-13
11	131.6, C			
12	25.6, CH_3	1.65 s		C-10, C-13
13	17.8, CH_3	1.55 s		C-10, C-12
14	36.1, CH_2	α 1.92 d (13.6)		C-1, C-6, C-8
		β 2.95 overlap		
15	19.3, CH_3	1.43 s		C-1, C-8
1-OH		3.93 s		C-6
2-OCH_3/OH		4.50 brs		
3-OH		5.61 brs		
6-OH		4.16 s		C-1, C-5, C-6, C-14
7-OH		3.34		

Table S4. ^{1}H, ^{13}C, COSY, HMBC Spectroscopic Data for Compound **3** in $CDCl_3$.

no.	3		COSY	HMBC
	δ_C[a]	δ_H[b], mult (J in Hz)		
1	82.4, C			
2	87.4, CH	4.67, s		
3	207.7, C			
4	36.7, CH_2	α 2.78 td (13.7, 6.9)	H-5	C-2, C-3, C-6
		β 2.40 ddd (13.7, 5.4, 1.7)		
5	35.4, CH_2	α 1.72 ddd (13.5, 6.9, 1.7)	H-4	C-1, C-3, C-6
		β 2.26 td (13.5, 5.4)		
6	73.1, C			
7	76.8, C			
8	47.5, CH	2.91 dd (11.5, 2.5)	H-9	
9	26.6, CH_2	α 1.85 ddd (14.8, 11.5, 7.9)	H-8, H-10	C-11
		β 2.63 dd (15.5, 6.3)		
10	121.8, CH	5.22 t (7.3)	H-9	
11	134.1, C			
12	26.0, CH_3	1.75 s		C-10, C-11, C-13
13	18.1, CH_3	1.64 s,		C-10, C-11, C-12
14	37.5, CH_2	α 2.20 d (13.9)		C-1, C-6, C-8
		β 3.27 d (13.9)		
15	19.9, CH_3	1.54 s		C-7, C-8
1-OH				
2-OCH_3/OH	59.3, CH_3	3.55 s		C-2
6-OH		3.71 s		C-1, C-6
7-OH		4.28 s		C-7, C-8
