# Supplementary material for: Pseudallenes A and B, new sulfur-containing ovalicin sesquiterpenoid derivatives with antimicrobial activity from the deep-sea cold seep sediment-derived fungus Pseudallescheria boydii CS-793
Source: Beilstein J Org Chem. 2024 Feb 28;20:470–8. doi: 10.3762/bjoc.20.42 (PMC10910587; doi:10.3762/bjoc.20.42)

checkCIF/PLATON report

Structure factors have been supplied for datablock(s) t_a

THIS REPORT IS FOR GUIDANCE ONLY. IF USED AS PART OF A REVIEW PROCEDURE FOR PUBLICATION, IT SHOULD NOT REPLACE THE EXPERTISE OF AN EXPERIENCED CRYSTALLOGRAPHIC REFEREE.

No syntax errors found. CIF dictionary Interpreting this report

Datablock: t_a

Bond precision: C-C = 0.0072 A Wavelength=1.54178

Cell:	a=15.0176(3)	b=24.2644(4)	c=29.3542(5)
	alpha=90	beta=90	gamma=90
Temperature:	173 K		

	Calculated	Reported
Volume	10696.5(3)	10696.5(3)
Space group	P 21 21 21	P 21 21 21
Hall group	P 2ac 2ab	P 2ac 2ab
Moiety formula	C16 H26 O5 S, 5(C16 H28 O5 S)	C16 H26 O5 S, 5(C16 H28 O5 S)
Sum formula	C96 H166 O30 S6	C96 H166 O30 S6
Mr	1992.65	1992.64
Dx,g cm-3	1.237	1.237
Z	4	4
Mu (mm-1)	1.783	1.783
F000	4312.0	4312.0
F000'	4332.48	
h,k,lmax	18,29,35	17,29,35
Nref	19580[10635]	19396
Tmin,Tmax	0.762,0.807	0.568,0.753
Tmin'	0.691	

Correction method= # Reported T Limits: Tmin=0.568 Tmax=0.753
AbsCorr = MULTI-SCAN

Data completeness= 1.82/0.99 Theta(max)= 68.182

R(reflections)= 0.0512(17030) wR2(reflections)= 0.1430(19396)

S = 1.000 Npar= 1252

The following ALERTS were generated. Each ALERT has the format
test-name_ALERT_alert-type_alert-level.
Click on the hyperlinks for more details of the test.

Alert level C

PLAT094_ALERT_2_C Ratio of Maximum / Minimum Residual Density .... 2.10 Report
PLAT220_ALERT_2_C NonSolvent Resd 1 C Ueq(max)/Ueq(min) Range 4.4 Ratio
PLAT220_ALERT_2_C NonSolvent Resd 2 C Ueq(max)/Ueq(min) Range 3.2 Ratio
PLAT220_ALERT_2_C NonSolvent Resd 5 C Ueq(max)/Ueq(min) Range 3.2 Ratio
PLAT222_ALERT_3_C NonSolvent Resd 1 H Uiso(max)/Uiso(min) Range 5.0 Ratio
PLAT230_ALERT_2_C Hirshfeld Test Diff for C12 --C13 . 5.2 s.u.
PLAT234_ALERT_4_C Large Hirshfeld Difference C76 --C77A . 0.16 Ang.
PLAT241_ALERT_2_C High 'MainMol' Ueq as Compared to Neighbors of C76 Check
PLAT242_ALERT_2_C Low 'MainMol' Ueq as Compared to Neighbors of C78 Check
PLAT242_ALERT_2_C Low 'MainMol' Ueq as Compared to Neighbors of C14 Check
PLAT242_ALERT_2_C Low 'MainMol' Ueq as Compared to Neighbors of C30 Check
PLAT242_ALERT_2_C Low 'MainMol' Ueq as Compared to Neighbors of C62 Check
PLAT340_ALERT_3_C Low Bond Precision on C-C Bonds .... 0.00718 Ang.
PLAT411_ALERT_2_C Short Inter H...H Contact H35B ..H83A . 2.12 Ang.
1+x,y,z = 1_655 Check
PLAT413_ALERT_2_C Short Inter XH3 .. XHn H50B ..H79A . 2.06 Ang.
-x,1/2+y,1/2-z = 3_555 Check
PLAT601_ALERT_2_C Unit Cell Contains Solvent Accessible VOIDS of . 47 Ang**3
PLAT767_ALERT_4_C INS Embedded LIST 6 Instruction Should be LIST 4 Please Check
PLAT911_ALERT_3_C Missing FCF Refl Between Thmin & STh/L= 0.600 26 Report
PLAT975_ALERT_2_C Check Calcd Resid. Dens. 0.97Ang From C68 . 0.42 eA-3
PLAT987_ALERT_1_C The Flack x is >> 0 - Do a BASF/TWIN Refinement Please Check

Alert level G

PLAT007_ALERT_5_G Number of Unrefined Donor-H Atoms .... 22 Report
PLAT033_ALERT_4_G Flack x Value Deviates > 3.0 * sigma from Zero . 0.019 Note
PLAT301_ALERT_3_G Main Residue Disorder ....(Resd 1) 5% Note
PLAT343_ALERT_2_G Unusual sp? Angle Range in Main Residue for C68 Check
PLAT367_ALERT_2_G Long? C(sp?)-C(sp?) Bond C67 - C68 . 1.52 Ang.
PLAT367_ALERT_2_G Long? C(sp?)-C(sp?) Bond C68 - C69 . 1.54 Ang.
PLAT367_ALERT_2_G Long? C(sp?)-C(sp?) Bond C78 - C79 . 1.62 Ang.
PLAT773_ALERT_2_G Check long C-C Bond in CIF: C77A --C79 1.89 Ang.
PLAT779_ALERT_4_G Suspect or Irrelevant (Bond) Angle(s) in CIF ... 41.40 Deg.
C78 -C79 -C77A 1_555 1_555 1_555 ...... # 181 Check
PLAT791_ALERT_4_G Model has Chirality at C1 (Sohnke SpGr) R Verify
PLAT791_ALERT_4_G Model has Chirality at C2 (Sohnke SpGr) R Verify
PLAT791_ALERT_4_G Model has Chirality at C3 (Sohnke SpGr) R Verify
PLAT791_ALERT_4_G Model has Chirality at C6 (Sohnke SpGr) R Verify
PLAT791_ALERT_4_G Model has Chirality at C8 (Sohnke SpGr) S Verify
PLAT791_ALERT_4_G Model has Chirality at C9 (Sohnke SpGr) R Verify
PLAT791_ALERT_4_G Model has Chirality at C17 (Sohnke SpGr) R Verify
PLAT791_ALERT_4_G Model has Chirality at C18 (Sohnke SpGr) R Verify
PLAT791_ALERT_4_G Model has Chirality at C19 (Sohnke SpGr) R Verify
PLAT791_ALERT_4_G Model has Chirality at C22 (Sohnke SpGr) R Verify
PLAT791_ALERT_4_G Model has Chirality at C24 (Sohnke SpGr) S Verify
PLAT791_ALERT_4_G Model has Chirality at C25 (Sohnke SpGr) R Verify
PLAT791_ALERT_4_G Model has Chirality at C33 (Sohnke SpGr) R Verify
PLAT791_ALERT_4_G Model has Chirality at C36 (Sohnke SpGr) R Verify
PLAT791_ALERT_4_G Model has Chirality at C37 (Sohnke SpGr) R Verify

```
PLAT791_ALERT_4_G Model has Chirality at C38         (Sohnke SpGr)          R Verify
PLAT791_ALERT_4_G Model has Chirality at C39         (Sohnke SpGr)          R Verify
PLAT791_ALERT_4_G Model has Chirality at C40         (Sohnke SpGr)          S Verify
PLAT791_ALERT_4_G Model has Chirality at C49         (Sohnke SpGr)          R Verify
PLAT791_ALERT_4_G Model has Chirality at C52         (Sohnke SpGr)          R Verify
PLAT791_ALERT_4_G Model has Chirality at C53         (Sohnke SpGr)          R Verify
PLAT791_ALERT_4_G Model has Chirality at C54         (Sohnke SpGr)          R Verify
PLAT791_ALERT_4_G Model has Chirality at C56         (Sohnke SpGr)          S Verify
PLAT791_ALERT_4_G Model has Chirality at C57         (Sohnke SpGr)          R Verify
PLAT791_ALERT_4_G Model has Chirality at C65         (Sohnke SpGr)          R Verify
PLAT791_ALERT_4_G Model has Chirality at C69         (Sohnke SpGr)          R Verify
PLAT791_ALERT_4_G Model has Chirality at C70         (Sohnke SpGr)          R Verify
PLAT791_ALERT_4_G Model has Chirality at C71         (Sohnke SpGr)          R Verify
PLAT791_ALERT_4_G Model has Chirality at C81         (Sohnke SpGr)          R Verify
PLAT791_ALERT_4_G Model has Chirality at C84         (Sohnke SpGr)          R Verify
PLAT791_ALERT_4_G Model has Chirality at C85         (Sohnke SpGr)          R Verify
PLAT791_ALERT_4_G Model has Chirality at C86         (Sohnke SpGr)          R Verify
PLAT791_ALERT_4_G Model has Chirality at C87         (Sohnke SpGr)          R Verify
PLAT791_ALERT_4_G Model has Chirality at C88         (Sohnke SpGr)          S Verify
PLAT883_ALERT_1_G No Info/Value for _atom_sites_solution_primary .     Please Do !
PLAT912_ALERT_4_G Missing # of FCF Reflections Above STh/L=  0.600         17 Note
PLAT913_ALERT_3_G Missing # of Very Strong Reflections in FCF ....          2 Note
PLAT933_ALERT_2_G Number of HKL-OMIT Records in Embedded .res File          4 Note
PLAT965_ALERT_2_G The SHELXL WEIGHT Optimisation has not Converged     Please Check
PLAT978_ALERT_2_G Number C-C Bonds with Positive Residual Density.          0 Info
PLAT992_ALERT_5_G Repd & Actual _reflns_number_gt Values Differ by          4 Check
```

```
   0 ALERT level A = Most likely a serious problem - resolve or explain
   0 ALERT level B = A potentially serious problem, consider carefully
  20 ALERT level C = Check. Ensure it is not caused by an omission or oversight
  50 ALERT level G = General information/check it is not something unexpected

   2 ALERT type 1 CIF construction/syntax error, inconsistent or missing data
  22 ALERT type 2 Indicator that the structure model may be wrong or deficient
   5 ALERT type 3 Indicator that the structure quality may be low
  39 ALERT type 4 Improvement, methodology, query or suggestion
   2 ALERT type 5 Informative message, check
```

It is advisable to attempt to resolve as many as possible of the alerts in all categories. Often the minor alerts point to easily fixed oversights, errors and omissions in your CIF or refinement strategy, so attention to these fine details can be worthwhile. In order to resolve some of the more serious problems it may be necessary to carry out additional measurements or structure refinements. However, the purpose of your study may justify the reported deviations and the more serious of these should normally be commented upon in the discussion or experimental section of a paper or in the "special_details" fields of the CIF. checkCIF was carefully designed to identify outliers and unusual parameters, but every test has its limitations and alerts that are not important in a particular case may appear. Conversely, the absence of alerts does not guarantee there are no aspects of the results needing attention. It is up to the individual to critically assess their own results and, if necessary, seek expert advice.

Publication of your CIF in IUCr journals

A basic structural check has been run on your CIF. These basic checks will be run on all CIFs submitted for publication in IUCr journals (*Acta Crystallographica*, *Journal of Applied Crystallography*, *Journal of Synchrotron Radiation*); however, if you intend to submit to *Acta Crystallographica Section C* or *E* or *IUCrData*, you should make sure that full publication checks are run on the final version of your CIF prior to submission.

Publication of your CIF in other journals

Please refer to the *Notes for Authors* of the relevant journal for any special instructions relating to CIF submission.

PLATON version of 28/11/2022; check.def file version of 28/11/2022

Datablock t_a - ellipsoid plot

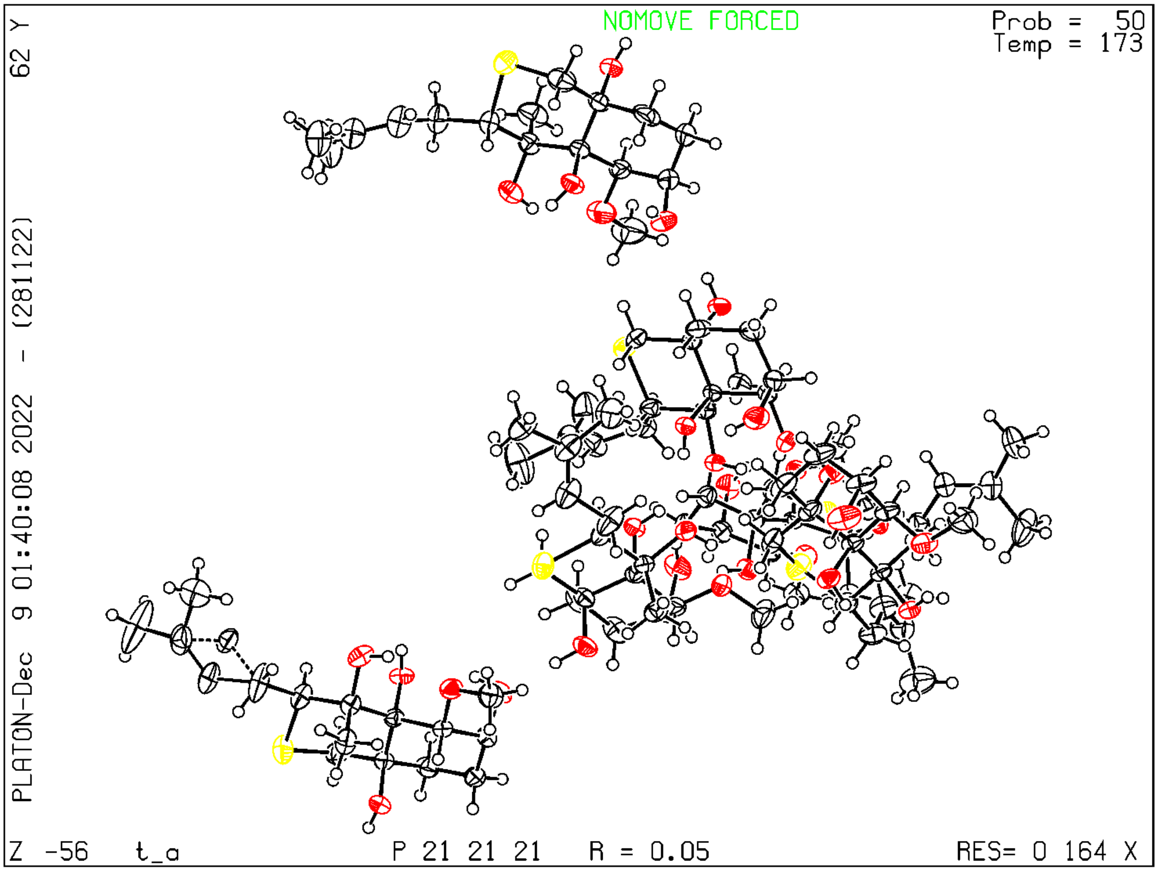

Supplement: File 2 — X-ray crystallographic files of compounds 1–3. [file Beilstein_J_Org_Chem-20-470-s002.zip › checkcif-cpd.1.pdf]
