# Supplementary material for: Pseudallenes A and B, new sulfur-containing ovalicin sesquiterpenoid derivatives with antimicrobial activity from the deep-sea cold seep sediment-derived fungus Pseudallescheria boydii CS-793
Source: Beilstein J Org Chem. 2024 Feb 28;20:470–8. doi: 10.3762/bjoc.20.42 (PMC10910587; doi:10.3762/bjoc.20.42)

checkCIF/PLATON report

Structure factors have been supplied for datablock(s) t

THIS REPORT IS FOR GUIDANCE ONLY. IF USED AS PART OF A REVIEW PROCEDURE FOR PUBLICATION, IT SHOULD NOT REPLACE THE EXPERTISE OF AN EXPERIENCED CRYSTALLOGRAPHIC REFEREE.

No syntax errors found. CIF dictionary Interpreting this report

Datablock: t

```
Bond precision:   C-C = 0.0047 A          Wavelength=1.54178

Cell:            a=6.5016(1)        b=7.7295(2)       c=17.5958(4)
                 alpha=85.427(1)    beta=81.799(1)    gamma=65.151(1)
Temperature:     173 K

                  Calculated                        Reported
Volume            794.00(3)                         794.00(3)
Space group       P 1                               P 1
Hall group        P 1                               P 1
Moiety formula    C15 H26 O5 S                      C15 H26 O5 S
Sum formula       C15 H26 O5 S                      C15 H26 O5 S
Mr                318.42                            318.42
Dx,g cm-3         1.332                             1.332
Z                 2                                 2
Mu (mm-1)         1.979                             1.979
F000              344.0                             344.0
F000'             345.67
h,k,lmax          7,9,20                            7,9,20
Nref              5612[ 2806]                       5468
Tmin,Tmax         0.735,0.789                       0.636,0.753
Tmin'             0.667

Correction method= # Reported T Limits: Tmin=0.636 Tmax=0.753
AbsCorr = MULTI-SCAN

Data completeness= 1.95/0.97        Theta(max)= 66.525

R(reflections)= 0.0287( 5118)                          wR2(reflections)=
                                                       0.0736( 5468)
S = 1.034                    Npar= 415
```

The following ALERTS were generated. Each ALERT has the format
test-name_ALERT_alert-type_alert-level.
Click on the hyperlinks for more details of the test.

Alert level C

```
PLAT089_ALERT_3_C Poor Data / Parameter Ratio (Zmax < 18) ........       6.76 Note
PLAT220_ALERT_2_C NonSolvent   Resd 1  C   Ueq(max)/Ueq(min) Range        3.5 Ratio
PLAT220_ALERT_2_C NonSolvent   Resd 2  C   Ueq(max)/Ueq(min) Range        3.8 Ratio
PLAT222_ALERT_3_C NonSolvent Resd 1  H   Uiso(max)/Uiso(min) Range        4.4 Ratio
PLAT222_ALERT_3_C NonSolvent Resd 2  H   Uiso(max)/Uiso(min) Range        4.8 Ratio
PLAT340_ALERT_3_C Low Bond Precision on  C-C Bonds ...............    0.00473 Ang.
PLAT414_ALERT_2_C Short Intra D-H..H-X       H39       ..H48      .      1.99 Ang.
                                                   x,y,z  =      1_555 Check
PLAT767_ALERT_4_C INS Embedded LIST 6 Instruction Should be LIST 4     Please Check
PLAT911_ALERT_3_C Missing FCF Refl Between Thmin & STh/L=    0.595          3 Report
PLAT987_ALERT_1_C The Flack x is >> 0 -  Do a BASF/TWIN Refinement     Please Check
```

Alert level G

```
PLAT033_ALERT_4_G Flack x Value Deviates > 3.0 * sigma from Zero .      0.048 Note
PLAT154_ALERT_1_G The s.u.'s on the Cell Angles are Equal ..(Note)      0.001 Degree
PLAT791_ALERT_4_G Model has Chirality at C5          (Sohnke SpGr)          S Verify
PLAT791_ALERT_4_G Model has Chirality at C6          (Sohnke SpGr)          R Verify
PLAT791_ALERT_4_G Model has Chirality at C7          (Sohnke SpGr)          R Verify
PLAT791_ALERT_4_G Model has Chirality at C8          (Sohnke SpGr)          R Verify
PLAT791_ALERT_4_G Model has Chirality at C12         (Sohnke SpGr)          S Verify
PLAT791_ALERT_4_G Model has Chirality at C13         (Sohnke SpGr)          R Verify
PLAT791_ALERT_4_G Model has Chirality at C20         (Sohnke SpGr)          S Verify
PLAT791_ALERT_4_G Model has Chirality at C21         (Sohnke SpGr)          R Verify
PLAT791_ALERT_4_G Model has Chirality at C22         (Sohnke SpGr)          R Verify
PLAT791_ALERT_4_G Model has Chirality at C23         (Sohnke SpGr)          R Verify
PLAT791_ALERT_4_G Model has Chirality at C29         (Sohnke SpGr)          R Verify
PLAT791_ALERT_4_G Model has Chirality at C30         (Sohnke SpGr)          S Verify
PLAT883_ALERT_1_G No Info/Value for _atom_sites_solution_primary .     Please Do !
PLAT909_ALERT_3_G Percentage of I>2sig(I) Data at Theta(Max) Still        88% Note
PLAT913_ALERT_3_G Missing # of Very Strong Reflections in FCF ....          2 Note
PLAT965_ALERT_2_G The SHELXL WEIGHT Optimisation has not Converged     Please Check
PLAT978_ALERT_2_G Number C-C Bonds with Positive Residual Density.          8 Info
```

  0 **ALERT level A** = Most likely a serious problem - resolve or explain
  0 **ALERT level B** = A potentially serious problem, consider carefully
 10 **ALERT level C** = Check. Ensure it is not caused by an omission or oversight
 19 **ALERT level G** = General information/check it is not something unexpected

  3 ALERT type 1 CIF construction/syntax error, inconsistent or missing data
  5 ALERT type 2 Indicator that the structure model may be wrong or deficient
  7 ALERT type 3 Indicator that the structure quality may be low
 14 ALERT type 4 Improvement, methodology, query or suggestion
  0 ALERT type 5 Informative message, check

It is advisable to attempt to resolve as many as possible of the alerts in all categories. Often the minor alerts point to easily fixed oversights, errors and omissions in your CIF or refinement strategy, so attention to these fine details can be worthwhile. In order to resolve some of the more serious problems it may be necessary to carry out additional measurements or structure refinements. However, the purpose of your study may justify the reported deviations and the more serious of these should normally be commented upon in the discussion or experimental section of a paper or in the "special_details" fields of the CIF. checkCIF was carefully designed to identify outliers and unusual parameters, but every test has its limitations and alerts that are not important in a particular case may appear. Conversely, the absence of alerts does not guarantee there are no aspects of the results needing attention. It is up to the individual to critically assess their own results and, if necessary, seek expert advice.

Publication of your CIF in IUCr journals

A basic structural check has been run on your CIF. These basic checks will be run on all CIFs submitted for publication in IUCr journals (*Acta Crystallographica*, *Journal of Applied Crystallography*, *Journal of Synchrotron Radiation*); however, if you intend to submit to *Acta Crystallographica Section C* or *E* or *IUCrData*, you should make sure that full publication checks are run on the final version of your CIF prior to submission.

Publication of your CIF in other journals

Please refer to the *Notes for Authors* of the relevant journal for any special instructions relating to CIF submission.

PLATON version of 28/11/2022; check.def file version of 28/11/2022

Datablock t - ellipsoid plot

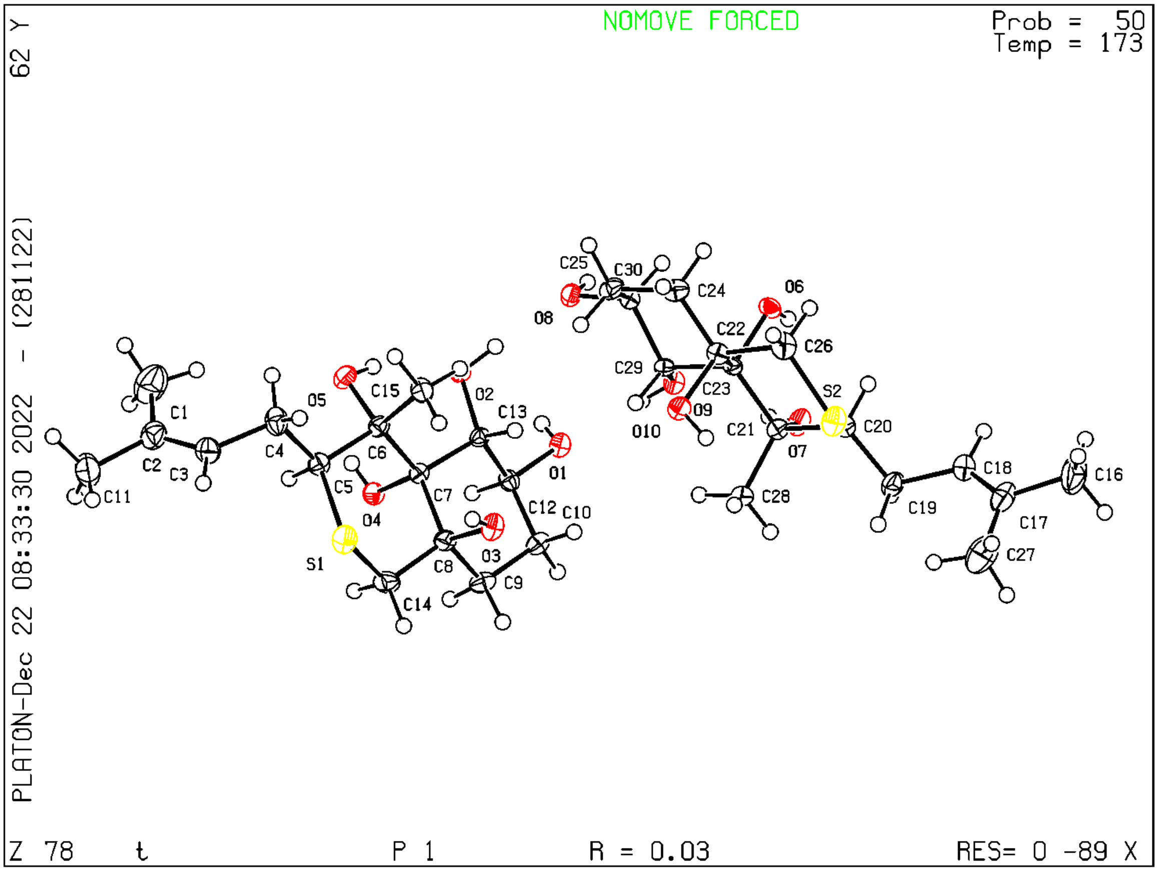

Supplement: File 2 — X-ray crystallographic files of compounds 1–3. [file Beilstein_J_Org_Chem-20-470-s002.zip › checkcif-cpd.2.pdf]
