# Supplementary material for: Pseudallenes A and B, new sulfur-containing ovalicin sesquiterpenoid derivatives with antimicrobial activity from the deep-sea cold seep sediment-derived fungus Pseudallescheria boydii CS-793
Source: Beilstein J Org Chem. 2024 Feb 28;20:470–8. doi: 10.3762/bjoc.20.42 (PMC10910587; doi:10.3762/bjoc.20.42)

checkCIF/PLATON report

Structure factors have been supplied for datablock(s) t

THIS REPORT IS FOR GUIDANCE ONLY. IF USED AS PART OF A REVIEW PROCEDURE FOR PUBLICATION, IT SHOULD NOT REPLACE THE EXPERTISE OF AN EXPERIENCED CRYSTALLOGRAPHIC REFEREE.

No syntax errors found. CIF dictionary Interpreting this report

Datablock: t

```
Bond precision:   C-C = 0.0055 A          Wavelength=1.54178

Cell:              a=13.9963(1)      b=13.9963(1)      c=30.1665(6)
                   alpha=90          beta=90           gamma=120
Temperature:       173 K

                 Calculated                  Reported
Volume           5117.79(13)                 5117.78(13)
Space group      P 61                        P 61
Hall group       P 61                        P 61
Moiety formula   C16 H26 O5 S                C16 H26 O5 S
Sum formula      C16 H26 O5 S                C16 H26 O5 S
Mr               330.43                      330.43
Dx,g cm-3        1.287                       1.287
Z                12                          12
Mu (mm-1)        1.863                       1.863
F000             2136.0                      2136.0
F000'            2146.24
h,k,lmax         16,16,36                    16,16,36
Nref             6254[ 3191]                 6250
Tmin,Tmax        0.779,0.800                 0.602,0.753
Tmin'            0.707

Correction method= # Reported T Limits: Tmin=0.602 Tmax=0.753
AbsCorr = MULTI-SCAN

Data completeness= 1.96/1.00       Theta(max)= 68.266

R(reflections)= 0.0317( 5717)                          wR2(reflections)=
                                                       0.0883( 6250)

S = 0.947                    Npar= 442
```

The following ALERTS were generated. Each ALERT has the format
test-name_ALERT_alert-type_alert-level.
Click on the hyperlinks for more details of the test.

Alert level C

```
PLAT089_ALERT_3_C Poor Data / Parameter Ratio (Zmax < 18) ........       7.22 Note
PLAT220_ALERT_2_C NonSolvent   Resd 1  C   Ueq(max)/Ueq(min) Range        3.1 Ratio
PLAT220_ALERT_2_C NonSolvent   Resd 2  C   Ueq(max)/Ueq(min) Range        3.2 Ratio
PLAT241_ALERT_2_C High   'MainMol' Ueq as Compared to Neighbors of         C7 Check
PLAT241_ALERT_2_C High   'MainMol' Ueq as Compared to Neighbors of        C24 Check
PLAT242_ALERT_2_C Low    'MainMol' Ueq as Compared to Neighbors of         C8 Check
PLAT242_ALERT_2_C Low    'MainMol' Ueq as Compared to Neighbors of        C25 Check
PLAT340_ALERT_3_C Low Bond Precision on  C-C Bonds ..............    0.00546 Ang.
PLAT413_ALERT_2_C Short Inter XH3 .. XHn     H36      ..H43      .       2.06 Ang.
                                    1+y,1-x+y,-1/6+z  =      3_664 Check
PLAT767_ALERT_4_C INS Embedded LIST 6 Instruction Should be LIST 4     Please Check
PLAT767_ALERT_4_C INS Embedded LIST 6 Instruction Should be LIST 4     Please Check
PLAT911_ALERT_3_C Missing FCF Refl Between Thmin & STh/L=    0.600          3 Report
PLAT987_ALERT_1_C The Flack x is >> 0 -  Do a BASF/TWIN Refinement     Please Check
```

Alert level G

```
PLAT007_ALERT_5_G Number of Unrefined Donor-H Atoms .............          2 Report
PLAT033_ALERT_4_G Flack x Value Deviates > 3.0 * sigma from Zero .      0.024 Note
PLAT112_ALERT_2_G ADDSYM Detects New (Pseudo) Symm. Elem         2        100 %Fit
PLAT112_ALERT_2_G ADDSYM Detects New (Pseudo) Symm. Elem         2        100 %Fit
PLAT112_ALERT_2_G ADDSYM Detects New (Pseudo) Symm. Elem         2        100 %Fit
PLAT112_ALERT_2_G ADDSYM Detects New (Pseudo) Symm. Elem         2        100 %Fit
PLAT112_ALERT_2_G ADDSYM Detects New (Pseudo) Symm. Elem         2        100 %Fit
PLAT112_ALERT_2_G ADDSYM Detects New (Pseudo) Symm. Elem         2        100 %Fit
PLAT113_ALERT_2_G ADDSYM Suggests Possible Pseudo/New  Space Group      P6122 Check
              Check Model Parameter Symmmetry for Reflection Data Support
PLAT301_ALERT_3_G Main Residue  Disorder .............(Resd  1  )          5% Note
PLAT301_ALERT_3_G Main Residue  Disorder .............(Resd  2  )          5% Note
PLAT412_ALERT_2_G Short Intra XH3 .. XHn     H6       ..H9BB     .       2.08 Ang.
                                                  x,y,z  =       1_555 Check
PLAT412_ALERT_2_G Short Intra XH3 .. XHn     H13      ..H9BA     .       1.90 Ang.
                                                  x,y,z  =       1_555 Check
PLAT412_ALERT_2_G Short Intra XH3 .. XHn     H26A     ..H35      .       2.03 Ang.
                                                  x,y,z  =       1_555 Check
PLAT412_ALERT_2_G Short Intra XH3 .. XHn     H26C     ..H43      .       2.04 Ang.
                                                  x,y,z  =       1_555 Check
PLAT720_ALERT_4_G Number of Unusual/Non-Standard Labels .........          6 Note
PLAT791_ALERT_4_G Model has Chirality at C2         (Sohnke SpGr)          S Verify
PLAT791_ALERT_4_G Model has Chirality at C3         (Sohnke SpGr)          R Verify
PLAT791_ALERT_4_G Model has Chirality at C4         (Sohnke SpGr)          R Verify
PLAT791_ALERT_4_G Model has Chirality at C5         (Sohnke SpGr)          S Verify
PLAT791_ALERT_4_G Model has Chirality at C12        (Sohnke SpGr)          R Verify
PLAT791_ALERT_4_G Model has Chirality at C19        (Sohnke SpGr)          S Verify
PLAT791_ALERT_4_G Model has Chirality at C20        (Sohnke SpGr)          R Verify
PLAT791_ALERT_4_G Model has Chirality at C21        (Sohnke SpGr)          R Verify
PLAT791_ALERT_4_G Model has Chirality at C22        (Sohnke SpGr)          S Verify
PLAT791_ALERT_4_G Model has Chirality at C30        (Sohnke SpGr)          R Verify
PLAT883_ALERT_1_G No Info/Value for _atom_sites_solution_primary .     Please Do !
PLAT913_ALERT_3_G Missing # of Very Strong Reflections in FCF ....          2 Note
```

```
PLAT933_ALERT_2_G Number of HKL-OMIT Records in Embedded .res File          1 Note
PLAT965_ALERT_2_G The SHELXL WEIGHT Optimisation has not Converged     Please Check
PLAT978_ALERT_2_G Number C-C Bonds with Positive Residual Density.         14 Info
```

```
   0 ALERT level A = Most likely a serious problem - resolve or explain
   0 ALERT level B = A potentially serious problem, consider carefully
  13 ALERT level C = Check. Ensure it is not caused by an omission or oversight
  31 ALERT level G = General information/check it is not something unexpected

   2 ALERT type 1 CIF construction/syntax error, inconsistent or missing data
  21 ALERT type 2 Indicator that the structure model may be wrong or deficient
   6 ALERT type 3 Indicator that the structure quality may be low
  14 ALERT type 4 Improvement, methodology, query or suggestion
   1 ALERT type 5 Informative message, check
```

It is advisable to attempt to resolve as many as possible of the alerts in all categories. Often the minor alerts point to easily fixed oversights, errors and omissions in your CIF or refinement strategy, so attention to these fine details can be worthwhile. In order to resolve some of the more serious problems it may be necessary to carry out additional measurements or structure refinements. However, the purpose of your study may justify the reported deviations and the more serious of these should normally be commented upon in the discussion or experimental section of a paper or in the "special_details" fields of the CIF. checkCIF was carefully designed to identify outliers and unusual parameters, but every test has its limitations and alerts that are not important in a particular case may appear. Conversely, the absence of alerts does not guarantee there are no aspects of the results needing attention. It is up to the individual to critically assess their own results and, if necessary, seek expert advice.

Publication of your CIF in IUCr journals

A basic structural check has been run on your CIF. These basic checks will be run on all CIFs submitted for publication in IUCr journals (*Acta Crystallographica*, *Journal of Applied Crystallography*, *Journal of Synchrotron Radiation*); however, if you intend to submit to *Acta Crystallographica Section C* or *E* or *IUCrData*, you should make sure that full publication checks are run on the final version of your CIF prior to submission.

Publication of your CIF in other journals

Please refer to the *Notes for Authors* of the relevant journal for any special instructions relating to CIF submission.

PLATON version of 28/11/2022; check.def file version of 28/11/2022

Datablock t - ellipsoid plot

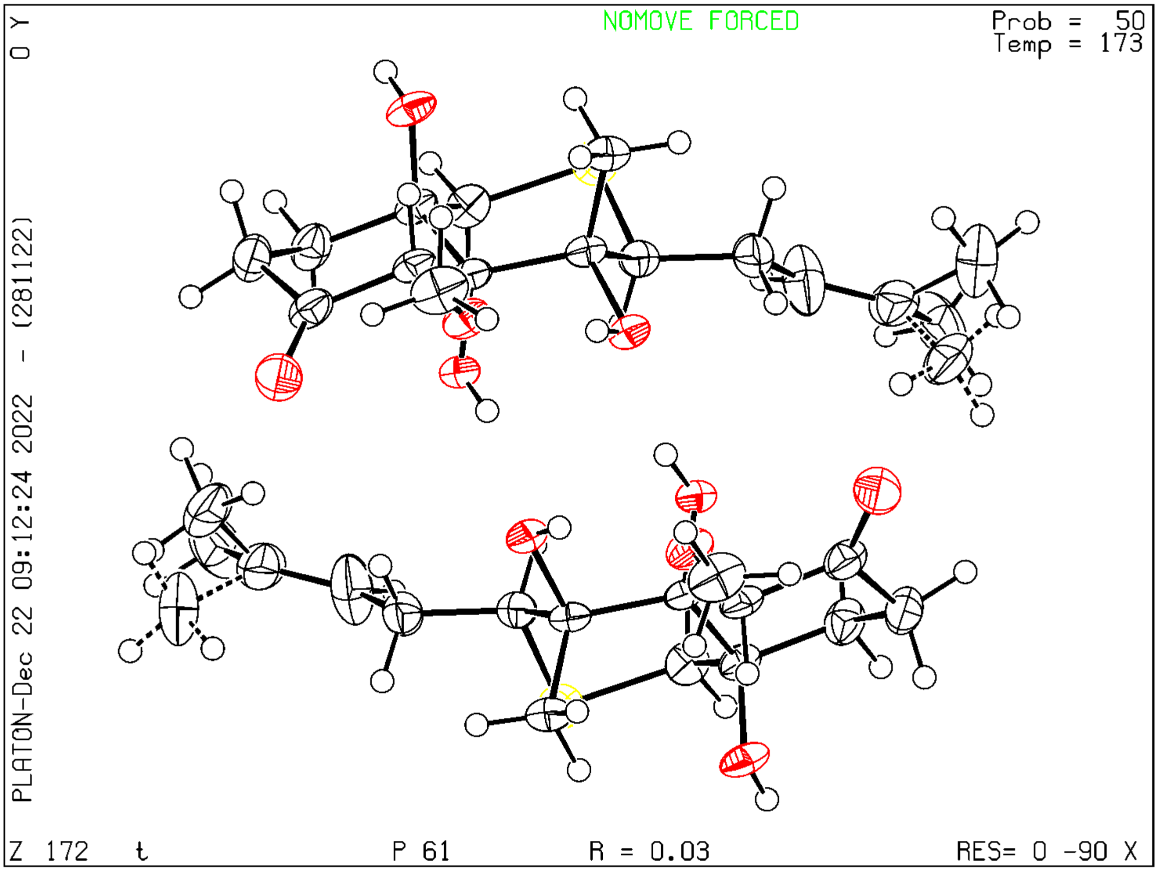

Supplement: File 2 — X-ray crystallographic files of compounds 1–3. [file Beilstein_J_Org_Chem-20-470-s002.zip › checkcif-cpd.3.pdf]
